# Charge Scaling Force Field for Biologically Relevant Ions Utilizing a Global Optimization Method

**DOI:** 10.1021/acs.jctc.5c00873

**Published:** 2025-09-04

**Authors:** Shujie Fan, Philip E. Mason, Victor Cruces Chamorro, Brennon L. Shanks, Hector Martinez-Seara, Pavel Jungwirth

**Affiliations:** 48311 Institute of Organic Chemistry and Biochemistry, Academy of Sciences of the Czech Republic, Flemingovo nam. 2, Prague 6 CZ-16610, Czech Republic

## Abstract

Charge scaling, also denoted as the electronic continuum correction, has proven to be an efficient method for effectively including electronic polarization in force field molecular dynamics simulations without additional computational costs. However, scaling charges in existing force fields, fitted at least in part to experimental data, lead to inconsistencies, such as overscaling. We have, therefore, recently developed a four-site water model consistent with charge scaling, i.e., possessing the correct low-frequency dielectric constant of 45. Here, we build on top of this water model to develop charge-scaled models of biologically relevant Li^+^, Na^+^, K^+^, Ca^2+^, and Mg^2+^ cations as well as Cl^–^, Br^–^, and I^–^ anions, employing machine learning to streamline and speed up the parametrization process. On the one hand, we show that the present model outperforms the best existing charge scaled model of aqueous ions. On the other hand, the present work points to a future need for consistently and simultaneously improving the water and ion models within the electronic continuum correction framework.

## Graphical abstract

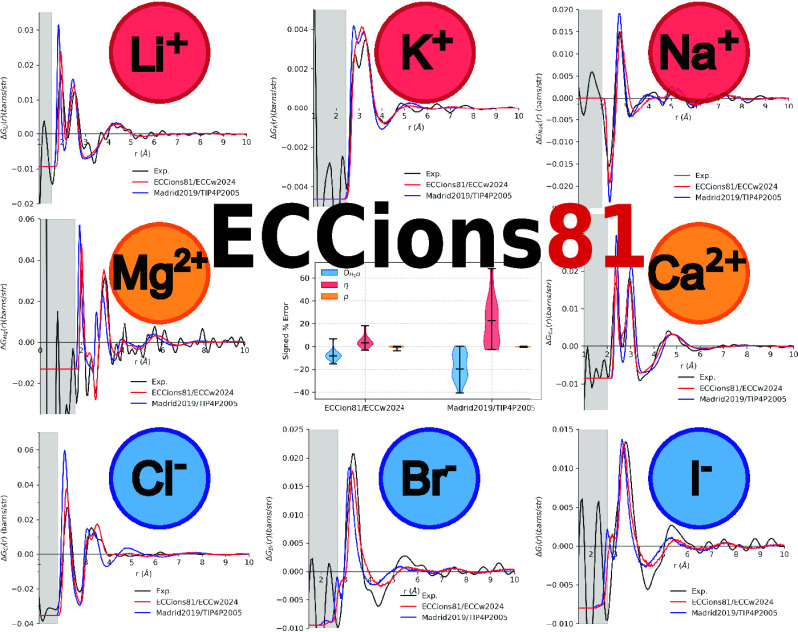

## Introduction

Effective inclusion of electronic polarization by charge scaling has been shown to improve the description of interactions of ions in aqueous environments without additional computational costs.^1–4^ This so-called electronic continuum correction (ECC) can, in particular, fix overbinding pertinent to high charge density ions like lithium, calcium, magnesium, and others described by standard nonpolarizable force fields.^5–8^ While this correction is well physically justified—it is equivalent to immersing the system into a dielectric continuum with the high-frequency dielectric constant (of about 1.78 for water)—one has to be careful not to overscale. Namely, existing nonpolarizable water models have been parametrized against experiment and, as a result, have dielectric constants higher than what would correspond purely to the dielectric response of the nuclei.^9–11^ In other words, (part of) the high-frequency electronic response has been translated in an uncontrolled way to low-frequency nuclear rearrangements. In order to avoid potential artifacts connected with grafting charge scaling on top of such water models, we have recently parametrized a *de novo* four-site water model better compatible with the ECC approach, i.e., possessing a dielectric constant of 45, corresponding to the genuine nuclear response only.^12^ We have shown that despite its low dielectric constant, this model, denoted as ECCw2024, performs well as the best existing four-site water models, such as TIP4P/2005.^11,12^

In this work, we take a step further in developing from scratch a charge-scaled force field for biomolecular simulations that is compatible from the onset with the ECC concept. Using machine learning techniques to save computational time during parametrization, we build parameters for the following biologically relevant ions, including Li^+^, Na^+^, K^+^, Ca^2+^, Mg^2+^, Cl^–^, Br^–^, and I^–^. By obtaining better agreement with experimental structural, thermodynamic, and especially dynamic properties for the corresponding salt solutions than the hitherto best model of this type,^13^ we demonstrate that our systematic approach to developing charge-scaled force fields pays off. Additionally, while these ion models are built upon ECCw2024 water, we show that they also perform rather well with TIP4P/2005, demonstrating the robustness of our approach. Finally, by optimizing the scaling parameter within physically justified margins, we pave the way for further improvements to charge-scaled force fields.

## Methods

### Optimization Process

An automated framework was utilized for the optimization process that was originally developed for parameter sampling of the ECC water model.^12^ A random walk algorithm was first applied to generate 200 parameter sets, with initial values taken from the prosECCo75 force field.^14^ Following this initial sampling, a differential evolution (DE) algorithm was used to refine the parameters and achieve an optimized data set.

Each sample was evaluated using a negative log-likelihood cost function defined as:

1 $$J\left(\theta \right)=\overset{n}{\underset{i=1}{\sum }}\left[\frac{n}{2}\log\left(2\pi \sigma_{i}^{2}\right)+\frac{1}{2\sigma_{i}^{2}}{\left(y_{i}(\theta )-\mu_{i}\right)}^{2}\right]$$

where **θ** denotes the parameter set, *y*_*i*_ represents the target property calculated from MD simulations, μ_*i*_ denotes the corresponding reference experimental value, σ_*i*_^2^ represents the predefined sample variance. In practice, all target properties were included during the optimization to achieve a globally optimized parameter set that performed well across multiple properties, rather than excelling at reproducing a single property.

To efficiently explore the parameter space while avoiding regions where predicted properties deviate significantly from experimental references, we integrated a local Gaussian process (LGP) model^15^ with the differential evolution algorithm. DE is a population-based global optimization method that evolves a set of candidate solutions (parameter vectors) through mutation, crossover, and selection. In each generation, new candidate parameter sets (offspring, denoted **θ**) are generated by perturbing existing ones (parents, denoted **θ**′), and a selection step determines whether the offspring should replace its parent.

To accelerate the evaluation of new parameter sets, the LGP model was used to predict physical properties directly, bypassing the need for full molecular dynamics (MD) simulations. The LGP model was initially trained on data generated by a random-walker algorithm, using force field parameters as input and corresponding target physical properties as output. As the optimization progressed, the LGP model was retrained after every 50 new data points, using the entire data set collected so far, including both existing and newly added examples. This active learning scheme ensured that the model continuously adapted to the expanding and increasingly diverse data space.

In practice, consider, for example, a parent parameter set **θ**′ that has already been evaluated through MD simulation, resulting in a cost function value *J*(**θ**′). A new offspring parameter set **θ** is generated by applying DE’s mutation and crossover steps to **θ**′. Before committing computational resources to simulate **θ**, the LGP model estimates the physical properties and computes a predicted cost *J*(**θ**). The selection of whether to accept **θ** is determined by the Metropolis–Hastings criterion:

2 $$A\left(\theta ,\theta^{\prime }\right)=\min\left(1,e^{J(\theta^{\prime })-J\left(\theta \right)}\right)$$

A random number *u* ∈ [0,1] is drawn, and if *u* ≤ *A*(**θ**, **θ**′), the offspring **θ** is accepted for MD simulation and added to the training data set; otherwise, it is rejected and discarded.

The parameters of the atomic ions were optimized against the target values, including structural information from neutron scattering, thermodynamic properties (e.g., density), and kinetic properties such as viscosity. Diffusion coefficients of water and oxygen in the solutions were also used for further validation. The atomic ions were divided into two groups based on the methods used to measure the structure of aqueous solutions. The first group, Li^+^, K^+^, Ca^2+^, and Cl^–^, was measured in solutions of 3 m LiCl,^6^ 6 m LiCl,^16^ 4 m KCl,^17^ and 4 m CaCl_2_ ^18^ using neutron diffraction experiments with isotopic substitution (NDIS). This method enables the estimation of partial structure factors from the total structure factor by assuming that solutions with identical chemical compositions but different isotopic concentrations are structurally equivalent. The second group, consisting of Na^+^, Mg^2+^, Br^–^, and I^–^, was measured in 4 m NaCl,^19^ 3 m MgCl_2_,^7^ 4 m KBr, and 4 m KI solutions using a “null” water mixture technique.^20^ The ions in the first group were optimized before those in the second group, as NDIS provides more detailed insights into ion-solution correlations.

The optimized parameters included the scaling factor and Lennard-Jones (LJ) ϵ and σ values for cations and anions, following the Lorentz–Berthelot combination rules:

3 $$\sigma_{ij}=\frac{\sigma_{ii}+\sigma_{jj}}{2},,\epsilon_{ij}=\sqrt{\epsilon_{ii}\epsilon_{jj}}$$

Furthermore, we included pair-specific LJ ϵ and σ parameters between anions and water–oxygen atoms as they improved the physical qualities of the ionic solutions. However, not all possible pairwise parameters (i.e., cation–anion, cation–oxygen, anion–oxygen, and self–self interactions) were explicitly included. This decision was based on two key considerations: 1. For the ionic solutions studied here, cation–cation and anion–anion LJ interactions have minimal impact on the target properties. Therefore, the meaningful interactions are primarily cation–anion, cation–oxygen, and anion–oxygen, and the degrees of freedom for the parameters of these interactions are 6 (3 × 2), where the 2 corresponds to ϵ and σ. This is equivalent to the degrees of freedom when considering only the combination rules ϵ and σ for cations, anions, and anion–oxygen pairs. 2. Including all pairwise interactions would significantly increase the dimensionality of the parameter space, resulting in higher computational costs for achieving convergence and generating sufficient samples to train the LGP model.

The parameters were initially sampled independently for each solution to explore the optimal model when there were no restrictions on either the scaling factor or the Cl^–^ model, which ultimately should be shared between salt pairs. During this procedure, each solution, such as LiCl, CaCl_2_, and KCl, was thus allowed to have different optimal scaling factors and Cl^–^ parameters. These separately optimized parameters served as training data for the LGP model and provided insight into the optimal behavior of ion models in a specific solution, which would help us determine whether it would be necessary to add cation–anion pairwise parameters during the final optimization. Then the Li^+^, Ca^2+^, K^+^, and Cl^–^ parameters were refined jointly, ensuring consistent Cl^–^ parameters and a shared scaling factor across all systems. We followed this by optimizing Na^+^, Mg^2+^, Br^–^, and I^–^ using the established optimal scaling factor and Cl^–^ parameters. I^–^ and Br^–^ were refined using a fixed scaling factor and the optimized K^+^ parameters from the previous stage, aiming to reproduce experimental behavior in 4 m of KI and KBr solutions. Similarly, Na^+^ and Mg^2+^ were optimized using the refined Cl^–^ model, with experimental data from 4 m NaCl and 3 m MgCl_2_ solutions as a reference. The optimization by stages was needed due to the differences in structural data (i.e., neutron scattering data) available for the salt solutions.

### Simulation Details

Molecular dynamics simulations were performed for all accepted parameters to estimate target properties, using the GROMACS 2022 molecular dynamics package.^21^ The simulated cubic box contained 2776 ECCw2024 water molecules, along with cations and anions, whose numbers were adjusted to achieve the target electrolyte concentration. Each system underwent energy minimization, followed by a 7 ns isothermal–isobaric (NPT) simulation. The NPT simulations were performed under periodic boundary conditions at a constant temperature (300 K for optimization and 298 K for validation) and pressure (1 bar). Temperature control was achieved using the Nosé–Hoover thermostat^22^ with a time constant of 1 ps, while an isotropic pressure coupling scheme was applied using the Parrinello–Rahman barostat^23^ with a compressibility of 5 × 10^–5^ bar^–1^ and a time constant of 5.0 ps. Long-range electrostatics and long-range Lennard-Jones potentials were calculated with the smooth particle mesh Ewald method^24^ with an initial cutoff of 1.2 nm. Interactions beyond the cutoff were calculated in reciprocal space with a fast-Fourier transform on a grid with an initial spacing of 0.10 nm and fourth-order spline interpolation.

Radial distribution functions (RDFs) and densities were calculated from the last 6 ns of the NPT simulation, with the initial 1 ns excluded for equilibration. The RDFs were calculated as

4 $$g_{\alpha \beta }\left(r\right)=\left(N_{\alpha }{N_{\beta })}^{-1}\overset{N_{\alpha }}{\underset{i=1}{\sum }}\overset{N_{\beta }}{\underset{j=1}{\sum }}\left\langle \delta (\right|r_{i}-r_{j}|-r\right)\rangle$$

where *N*_α_ and *N*_β_ are the total number of atom type α and atom type β, respectively. The calculated RDFs were compared with neutron scattering results, with additional details provided in the Supporting Information. To evaluate ion pairing in the first hydration shell of cations, the number of contact ion pairs (CIPs) was calculated from the cation–anion RDF as:

$$\mathrm{CIP}=4\pi \rho_{+/-}\left(r\right)\int_{0}^{r_{\min}}g_{+-}\left(r\right)r^{2}dr$$ 5

where *g*_+–_(*r*) is the RDF between cations and anions, and ρ_+/–_ is the number density of cations or anions. For 1:2 electrolytes, such as MgCl_2_ and CaCl_2_, we calculated CIPs using the anion’s number density ρ_–_ to ensure that the value reflects the number of anions in contact with the cation. The upper limit of the integral, *r*_min_, corresponds to the position of the first minimum in *g*_+–_(*r*). If no distinct peak is observed in *g*_+–_(*r*) within the first hydration shell, which is defined based on the ion–water RDF, CIP is considered to be zero. The hydration number (HN), representing the total number of water molecules in the first hydration shell, was computed based on the ion-oxygen RDF *g*_MO_:

$$HN^{O}=4\pi \rho_{H_{2}O}\left(r\right)\int_{0}^{r_{\min}^{MO}}g_{MO}\left(r\right)r^{2}dr$$ 6

where ρ_H2O_ is the number density of water molecules, and *r*_min_ corresponds to the first minimum in *g*_MO_. The total coordination number (CN) was then calculated as the sum of HN and CIP:

CN=HN+CIP 7

The final frame from the NPT simulation was used as the starting structure for three independent 2 ns nonequilibrium simulations with a cosine acceleration to calculate viscosity.^25^ The settings were identical to those of the NPT simulations, except that pressure coupling was disabled and a cosine acceleration amplitude of 0.020 nm/ps^2^ was applied.

To further evaluate the optimized models, additional simulations were performed with the optimal parameters. Equilibrium and nonequilibrium simulations were conducted for all salt solutions at concentrations of 0.24, 1, 3, and 4 M, with the same settings as those used during the optimization process. For MgCl_2_, which exhibits slow equilibration (see Supporting Information), 500 ns NPT simulations were conducted at the same concentrations to obtain converged properties for reproducibility. For LiCl, an additional concentration of 6 m was included. To ensure accurate estimates of means and standard deviations, 25 independent 2 ns nonequilibrium simulations with cosine acceleration were conducted for viscosity calculations. Similarly, 25 independent 2 ns NVT equilibrium simulations were performed to calculate the diffusion coefficient of water oxygen atoms in each solution.^26^ These NVT simulations followed the same settings as the NPT simulations, except that pressure coupling was disabled. The diffusion coefficient was calculated using the Einstein approach:

8 $$D=\underset{t\to \infty }{\lim}\frac{d}{dt}\left\langle \frac{1}{6N}\overset{N}{\underset{i=1}{\sum }}\right|r_{i}\left(t\right)-{r_{i}(0)|}^{2}\rangle$$

where *r*_*i*_(*t*) and *r*_*i*_(0) represent the position of the *i*th particle at time *t* and a reference time *t* = 0, respectively, and *N* is the total number of particles in the system. A finite-size correction was applied according to the method proposed by Yeh and Hummer:^27^

9 $$D_{\infty }=D\left(L\right)+\frac{k_{B}T\xi }{6\pi \eta L}$$

where *D*_∞_ is the infinite system size self-diffusion coefficient, *D*(*L*) is the self-diffusion coefficient calculated for a cubic box with edge length *L*, η is the shear viscosity obtained from the cosine acceleration nonequilibrium simulations, and ξ = 2.837298 is a dimensionless constant. It is important to note that this correction term incorporates simulation-derived parameters, which may introduce additional sources of error in estimating *D*_∞_. To assess this potential error, we compared the correction calculated using the edge length and shear viscosity of an ideal solution box, matching experimental density and viscosity values, and found that the additional error introduced by the simulation-derived *L* and η in the correction term was less than 2%. Therefore, we included the corrected results in our analysis.

We repeated the simulations for our optimal models using the TIP4P/2005 water model to test the transferability of our parameters to other water models. Additionally, simulations were carried out for the Madrid2019 ion models with both the ECCw2024 and TIP4P/2005 water models as references for comparison.

Additional 50 ns NPT simulations were conducted for 1 m solutions of LiCl, NaCl, KCl, CaCl_2_, and MgCl_2_ at a pressure of 1 bar over a temperature range of 233–298 K, in 5 K intervals, to determine the temperature of maximum density (TMD). The first 10 ns of each simulation represented equilibration, with densities calculated from the remaining trajectory. The resulting temperature-dependent densities were then fitted to a cubic polynomial, and the TMD was obtained as the temperature corresponding to the zero point of the first derivative of the fit.

## Results and Discussion

### Optimization of a ECC Compatible Ion Force Field

Building on the recently developed ECCw2024 water model, we now turn to the development of an ECC-compatible force field for ions. In this work, we systematically optimize the charge scaling factor and the Lennard-Jones (LJ) parameters σ and ϵ for a set of biologically and technologically relevant ions, namely, the Li^+^, Na^+^, K^+^, Ca^2+^, and Mg^2+^ cations and the Cl^–^, Br^–^, and I^–^ anions. To enhance the transferability of the resulting force field, we prioritize the use of combination rules over the inclusion of ion-specific pair interactions. Specific pair parameters are incorporated only when they yield clear and significant improvements in accuracy.

Our optimization method, targeting thermodynamic, dynamic, and structural properties, yielded a plethora of acceptable models, representing a sizable convex hull in parameter space. Figure 1 shows the distribution of scaling factors for all accepted samples during the optimization. These acceptable models exhibit scaling factors ranging from 0.7 to 0.9. The top-performing models have scaling factors between 0.78 and 0.83, indicating that good models are abundant within this range when optimized with our ECCw2024 water model. The scaling factor of 0.81 emerged as both the optimal and the most populated factor among the top 25 models ranked by the cost function.

**Figure 1:**
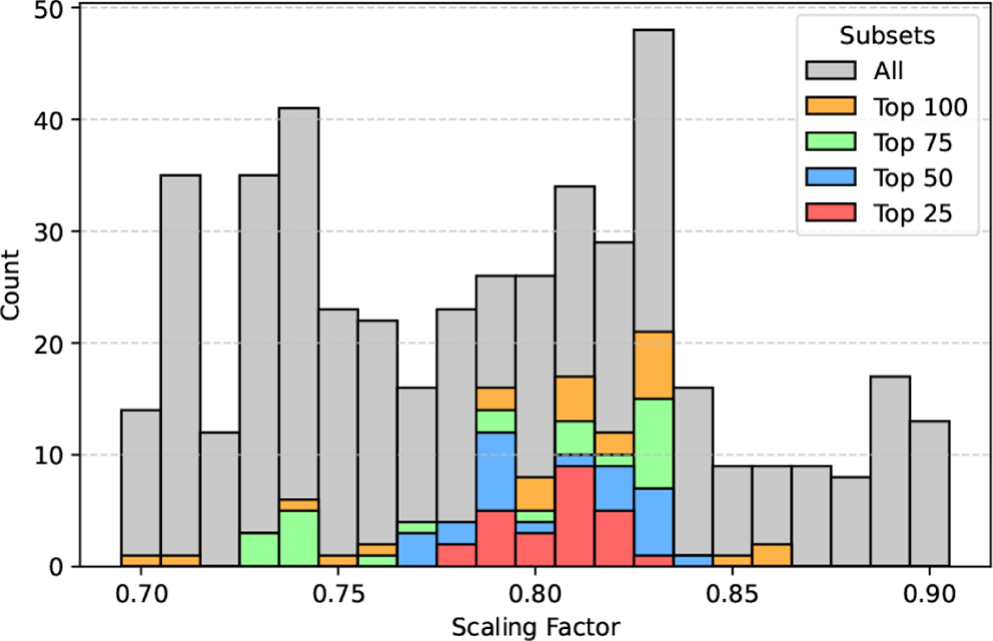
Distribution of scaling factors for the top 25, 50, 75, 100, and all samples from the final optimization stage of ions, including Li^+^, Na^+^, K^+^, Ca^2+^, and Cl^–^.

The fact that the optimal scaling factor of 0.81 is somewhat higher than the value of 0.75 following from the high-frequency dielectric constant of water is consistent with our recent finding of a slightly attenuated dielectric screening pertinent to closely interacting ions.^28^ Indeed, our optimization cost function incorporates neutron diffraction with isotopic substitution data (i.e., first-order differences in real-space signals), which primarily reflect the first and second hydration shells of solvated ions. Our optimal scaling factor also aligns closely with a recently developed scaled-charge potassium model with a scaling factor of 0.78, which shows excellent agreement with experimental conductivity data critical for simulating potassium channels.^29^

The set of parameters for the best-performing model, denoted as ECCions81, is presented in Table 1. It is of interest to compare these parameters with those of the Madrid2019 force field^13^ probably the best-performing charge-scaling ion model to date. Besides the difference in scaling factors (0.85 for Madrid2019), a notable distinction between the ECCions81 model and Madrid2019 lies in the resulting LJ parameters of our ions in water (Table S3). Cations in our ECCions81 model have systematically larger LJ σ values compared to Madrid2019, with an average increase of 0.17 Å for monovalent cations and 0.38 Å for divalent cations, reflecting a larger effective ionic radius in water. In contrast, our ECCions81 anions exhibit smaller σ values compared to Madrid2019, with an average reduction of 0.5 Å. Despite this reduction in σ, the anions still exhibit a larger effective ionic pair radius when compared to Madrid2019. Additionally, the difference between cation–oxygen and cation–anion σ values is significantly smaller in our ECCions81 model than in Madrid2019.

**Table 1:** Parameters of the ECCions81 Force Field^a^

Charges (*e*)					
*q*_Li_ = *q*_Na_ = *q*_K_ = 0.81, *q*_Mg_ = *q*_Ca_ = 1.62, *q*_Cl_ = *q*_Br_ = *q*_I_ = −0.81					

Ion	σ (nm)	ϵ (kJ/mol)	Ion	σ (nm)	ϵ (kJ/mol)
Li^+^	0.151568	0.217304	Cl^–^	0.393492	0.714723
Na^+^	0.237287	0.154682	Cl–O	0.352611	0.723797
K^+^	0.297000	0.844526	Br^–^	0.441421	1.515692
Mg^2+^	0.123364	4.504426	Br–O	0.360099	1.172294
Ca^2+^	0.240765	0.900759	I^–^	0.476172	1.295655
O	0.315480	0.761154	I–O	0.372802	0.921039

a Charges and Lennard-Jones σ and ϵ parameters for electrolyte solutions in ECCw2024 water are provided for Li^+^, Na^+^, K^+^, Mg^2+^, Ca^2+^, Cl^–^, Br^–^, and I^–^. Since the pairwise Lennard-Jones parameters between water oxygen and cations follow the Lorentz–Berthelot combination rules, the parameters for ECCw2024 oxygen are also listed.^12^ In contrast, specific anion–oxygen pairwise Lennard-Jones parameters are provided for anions.

These differences lead to a greater tendency for contact ion pairing (CIP) within the first hydration shell in the ECCions81 model, while the overall coordination number (HN_c_^O^ + CIP) remains nearly unchanged for cations compared to Madrid2019 (Table 2). The CIP values predicted by ECCions81 for LiCl, NaCl, and KBr are higher than those reported using the DLM/2022-BK3 polarizable force field^30,31^ with KI showing comparable values. Notably, DLM/2022-BK3 predicts no ion pairing for LiCl, while smaller CIP values are observed for the other salts. For anions, particularly at high concentrations where ion pairing is significant (e.g., 6 m LiCl, 4 m CaCl_2_), the hydration number calculated from the radial distribution function (RDF) between the anion and oxygen (HN_a_^O^) is influenced by the oxygen coordinating the contact pairing cations. We compared the hydrogen coordination numbers of anions (HN_a_^H^) for Cl^–^, Br^–^, and I^–^ at 1 M concentration using the ECCions81 force field against results from the DLM/2022-BK3 polarizable force field^31^ and hybrid QM/MM simulations^32–36^ (Table S4). Our analysis showed that the hydration coordination numbers calculated with ECCions81 were consistently close to but approximately 0.3 lower than those from DLM/2022-BK3. For HN_a_^O^, ECCions81 yielded values that were lower by about 1 compared to DLM/2022-BK3, yet still within the experimentally observed range. This increase in contact ion pairing affects radial distribution functions and significantly influences the transport and dynamic properties of solutions, which are explored in detail in the following sections.

**Table 2:** Comparison of CIP, HN Values of Cations (HN_c_^O^) or Anions (HN_a_^O^), Cation–Oxygen Distances, and Anion–Oxygen Distances for ECCions81, Madrid2019, and experiments^37–40^ across Various Salt Solutions

Solution	Model	CIP	${\mathrm{HN}}_{c}^{O}$	${\mathrm{HN}}_{a}^{O}$	$d_{c-O_{w}}$ (Å)	$d_{a-O_{w}}$ (Å)
6 m LiCl	ECCions81	0.43	3.60	7.38 (6.41)^a^	1.94	3.08
	Madrid2019	0	3.97	5.79	1.84	3.02
	Exp.		3.3–5.3	4–7.3	1.90–2.25	3.08–3.34
3 m LiCl	ECCions81	0.22	3.79	6.79	1.94	3.10
	Madrid2019	0	3.96	5.72	1.84	3.04
	Exp.		3.3–5.3	4–7.3	1.90–2.25	3.08–3.34
3 m MgCl_2_	ECCions81	0.01	6.05	6.38	2.02	3.10
	Madrid2019	0	5.90	5.76	1.92	3.04
	Exp.		6–8.1	6	2.00–2.11	3.13–3.16
4 m MgCl_2_	ECCions81	0.02	5.95	6.10	2.02	3.08
	Madrid2019	0	5.97	5.85	1.92	3.04
	Exp.		6–8.1	6	2.00–2.11	3.13–3.16
4 m CaCl_2_	ECCions81	1.04	6.35	8.06 (6.21)^a^	2.36	3.08
	Madrid2019	0.01	7.23	6.07	2.40	3.04
	Exp.		5.5–8.2	5.8–8.2	2.39–2.46	3.12–3.25
4 m KCl	ECCions81	0.80	5.85	5.22	2.80	3.08
	Madrid2019	0.10	6.52	5.79	2.72	3.04
	Exp.		6–8	6–8	2.60–2.80	3.08–3.16
4 m NaCl	ECCions81	0.58	4.78	6.35	2.32	3.08
	Madrid2019	0.04	5.38	5.94	2.34	3.04
	Exp.		4–8	5.5–6	2.41–2.50	3.08–3.20
4 m KBr	ECCions81	0.91	6.40	6.14	2.80	3.28
	Madrid2019	0.02	6.29	5.81	2.72	3.14
	Exp.		6–8	4–6	2.60–2.80	3.01–3.45
4 m KI	ECCions81	0.52	6.37	6.18	2.80	3.38
	Madrid2019	0.03	6.51	5.96	2.72	3.28
	Exp.		6–8	4–6	2.60–2.80	3.01–3.45

a In systems with a high prevalence of contact ion pairs, the first minimum of *g*_ClO_ may shift to larger distances, including the oxygen in the first hydration shell of the contact cation. To enable consistent comparison across concentrations, we report the anion hydration numbers in these cases using an *r*_min_ derived from dilute conditions (1 m), indicated in parentheses following each reported value.

### Densities and Dynamic Properties

Our ECCions81 ion model demonstrates excellent agreement with experimental density values, with deviations typically below 0.5%, comparable to the performance of Madrid2019 (Figure 2 and Table S5). This high level of accuracy is consistently observed across most tested solutions, including LiCl, NaCl, KI, and MgCl_2_, where both models align closely with the experimental measurements. In KCl and KBr solutions, ECCions81 slightly underestimates the density by approximately 0.7%, equivalent to a difference of ∼10 kg/m^3^. A more notable deviation occurs in CaCl_2_ solutions, where the ECCions81 model underestimates the density by ∼3.5% at 4 m, corresponding to a difference of ∼50 kg/m^3^ from the experimental values. Note that Madrid2019 accurately reproduces the experimental densities for these solutions. The temperatures of maximum density (TMD) for 1 m solutions of LiCl, NaCl, KCl, CaCl_2_, and MgCl_2_ were compared to experimental values^41–43^ and results of Madrid2019 ^42^ (Table S8). ECCions81 demonstrates good accuracy for LiCl, NaCl, and KCl solutions with TMD deviations below 3% and corresponding maximum density values deviating by less than 0.3%. For CaCl_2_, ECCions81 showed a ∼4% deviation in TMD and 1% in maximum density. This correlates with the above-discussed relatively less accurate density prediction for 4 m CaCl_2_ solutions.

**Figure 2:**
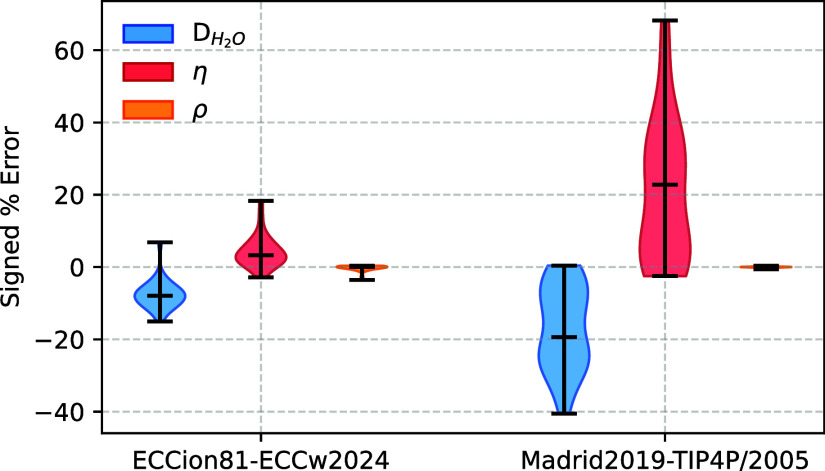
Signed percentage errors in the self-diffusion coefficient of water (*D*_H2O_), viscosity (η), and density (ρ) predicted from simulations using two water–ion models: ECCions81-ECCw2024 and Madrid2019-TIP4P/2005, with experimental data as the reference. Violin plots represent the distribution of errors across all tested electrolyte solutions at various concentrations. Horizontal bars indicate the medians, and the full range of data is shown to highlight the variability and systematic deviations.

The ECCions81 model outperforms Madrid2019 in reproducing the concentration dependence of both viscosity and water self-diffusion coefficients (*D*_OW_) across all tested electrolyte solutions (Figure 2 and Tables S6 and S7). Viscosity deviations in ECCions81 generally remain below 10%, while Madrid2019 frequently overestimates viscosity by more than 25% at high concentrations. The discrepancy is particularly severe for MgCl_2_, where Madrid2019 deviates by over 60% at 4 m, in contrast to ECCions81, which closely matches the experimental data. In LiCl solutions, ECCions81 shows somewhat higher deviations in viscosity (up to 16% at 6 m) yet still significantly improves on Madrid2019, which deviates by 64%. For KCl, KBr, and KI, where viscosity changes only weakly with concentration, the ECCions81 model accurately captures the trend with deviations below 10%, whereas Madrid2019 overshoots by more than 30%. Despite not being an explicit optimization target, we observe similar trends for water diffusion (*D*_OW_). Namely, ECCions81 maintains deviations within 10% for most salts, while Madrid2019 underestimates water mobility, with errors reaching as much as 40%. Again, 6 m LiCl is the most extreme case, with deviations of 15% for ECCions81 and 41% for Madrid2019. ECCions81 also better captures the weak concentration dependence of *D*_OW_ in KCl, KBr, and KI solutions, whereas Madrid2019 consistently overestimates the effect of salt concentration on dynamic properties. It is worth noting that a modified version of Madrid2019, using scaling factors of 0.8 and 0.75, referred to as Madrid-Transport, accurately reproduces viscosities and diffusion coefficients of KCl and NaCl solutions.^44^ These findings are consistent with the global optimal scaling factor of 0.81 identified in our study, which was determined based on a broad set of target properties.

### Comparisons to Neutron Scattering Data

This section compares the simulation results of the first-order difference functions in the *r*-space and *Q*-space for Cl^–^ with neutron diffraction data (i.e., NDIS experiments). While both representations contain the same information, they emphasize different aspects: *Q*-space highlights peak positions related to phasing, whereas *r*-space facilitates the interpretation of peak heights and the overall understanding of the molecular surroundings of the ions.

Overall, we see that ECCions81 systematically matches better experimental peak heights in *r*-space and phases in *Q*-space, with Madrid2019 consistently overestimating peak intensities in *r*-space. ECCions81 also improves the peak positions for Br^–^ and I^–^ and significantly reduces the artificial O–O peak at 2.8 Å (Figures S19 and S21). These improvements stem from moderate ion pairing in ECCions81, which weakens ion–water correlations.

Below, we focus on detailed comparisons between the simulation results and neutron diffraction experiments. We primarily discuss first-order difference functions (in both real and reciprocal spaces) where we possess high-quality experimental data for Li^+^, K^+^, Ca^2+^, and Cl^–^. Less direct neutron diffraction data are available to us for Mg^2+^, Br^–^, I^–^ and Na^+^ ions, as discussed briefly below and further in the Supporting Information.

#### Potassium

Monovalent ions, particularly the relatively large K^+^ ions that exhibit a low charge density, are generally considered straightforward to model. In principle, their weakly bound coordination shells should not pose a major challenge for classical force fields, and charge scaling is expected to play only a minor role in accurately capturing their solvation behavior. The comparison between simulation results and NDIS results for a 4 m KCl solution is shown in Figure 3. In R-space, Madrid2019 captures the successive dual peaks between 2.8 and 3.8 Å but significantly overestimates the height of the first peak, corresponding to the K–O correlation (Figure S8). In contrast, ECCions81 produces a single peak rather than dual peaks with an overshooting intensity similar to that of the Madrid model, while the first minimum fits the experimental data perfectly. Beyond this minimum, the apparent match with the experimental data is reduced with a significant diphase. In *Q*-space, both models exhibit a similar structure, characterized by higher first and second peaks, deeper minima between these peaks, and a reduced signal in the low-*Q* region compared with experimental values. While the low-*Q* signal for Madrid2019 is slightly higher than that of ECCions81, both models deviate from the experimental data in this region.

**Figure 3:**
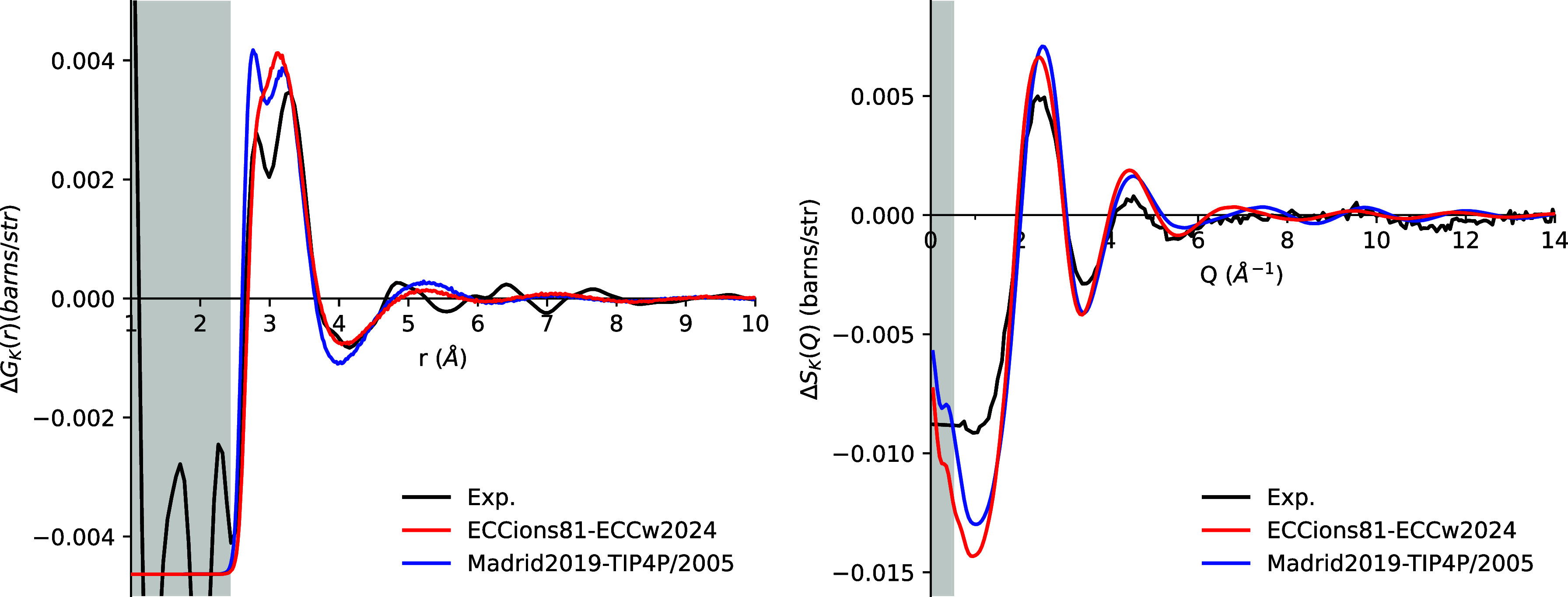
First-order difference functions Δ*G*_K_(*r*) (left) and Δ*S*_K_(*Q*) (right) for simulations of 4 m KCl solution using the ECCions81 model with the ECCw2024 water model (red), the Madrid2019 ion model with the TIP4P/2005 water model (blue), and experimental data (black). Gray shading indicates regions where the experimental signal consists of only noise (in *r*-space) or falls outside the measurement range (in *Q*-space).

A more detailed comparison of K^+^ simulation results with experimental data is provided in the Supporting Information, including a reference model that shows better agreement with the *r*-space data but yields a less accurate density. Overall, despite the anticipated simplicity in the description of potassium ions, none of the existing models fit the experimental data perfectly. Still, ECCions81 improves structural agreement compared to Madrid2019 while maintaining reasonable density predictions.

#### Calcium

Divalent cations with high-density charges are known to be harder to model, with charge scaling playing a more relevant role.^4^ First-order difference functions of Ca^2+^ in a 4 m CaCl_2_ solution in both *r*-space and *Q*-space were calculated and compared with experimental data, with the results shown in Figure 4. In *r*-space, both ECCions81 and Madrid2019 models capture the two prominent peaks at approximately 2.3 and 3.0 Å, as well as the third minor peak at around 4.8 Å. The first two peaks correspond to the Ca–O and Ca–H correlations in the first hydration shell. In contrast, the third peak arises from a combination of Ca–O, Ca–H, and Ca–Cl correlations in the second hydration shell, as shown in Figure S7. The stronger Ca–Cl pairing in ECCions81 reduces the correlations between Ca and water, introduces a small bump at the local minimum of around 2.6 Å, and lowers the height of the first and second peaks, bringing them closer to experimental values. In contrast, the Madrid2019 model does not exhibit any Ca–Cl pairing.

**Figure 4:**
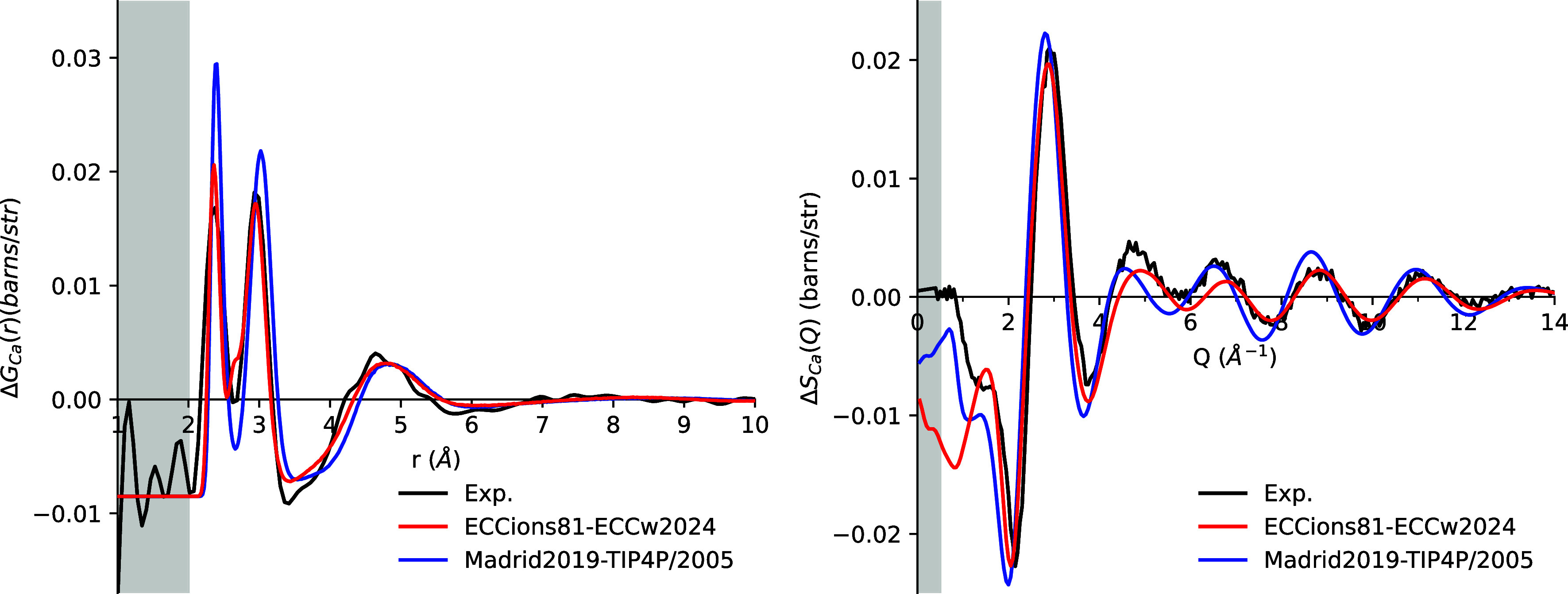
First-order difference functions Δ*G*_Ca_(*r*) (left) and Δ*S*_Ca_(*Q*) (right) for simulations of 4 m CaCl_2_ solution using the ECCions81 model with the ECCw2024 water model (red), the Madrid2019 ion model with the TIP4P/2005 water model (blue), and experimental data (black). Gray shading indicates regions where the experimental signal consists of only noise (in *r*-space) or falls outside the measurement range (in *Q*-space).

In *Q*-space, ECCions81 shows better overall agreement with experimental data than Madrid2019 across the full *Q*-range, particularly in capturing signal amplitudes. Madrid2019 exhibits slightly better phase agreement around 1 Å^–1^—a region corresponding to long-range structural correlations in *r*-space, which may be related to its improved performance in reproducing bulk density. It also shows improved phase alignment in the 4–7 Å^–1^ range, although the associated structural features in *r*-space are less clearly defined.

#### Lithium

Lithium represents a special case among monovalent ions due to its small size, resulting in a large charge density and, consequently, in a well-defined first coordination shell. The hydration structure of Li^+^ was obtained in 3 and 6 m LiCl solutions, with comparisons between simulation and experimental data as shown in Figures 5 and S4. In *r*-space, both the ECCions81 and Madrid2019 models roughly reproduce the structure of the signals at both concentrations. Two closely spaced peaks represent the Li–O and Li–H correlations, respectively, while a third broader peak arises from a mixture of Li–O, Li–H, and Li–Cl correlations (Figures S3 and S5). The Madrid2019 model overestimates the Li–O and Li–H correlations, with the Li–O peak approximately 50% higher than the experimental value at 3 m and 100% higher at 6 m, indicating a tighter first hydration shell. In contrast, the ECCions81 model correctly lowers the Li–O and Li–H peaks. While the LiO peak still remains somewhat higher than the experimental value, the Li–H peak aligns closely with the experimental data. The difference between the two force fields arises from the larger σ_Li–O_ in ECCions81 compared to Madrid2019 (Table S3), which also explains why the first peak of the ECCions81 model begins at a greater distance. Li–Cl pairing in ECCions81 leads to a reduction in the water density in the first hydration shell. In *Q*-space, ECCions81 shows better agreement with experimental data than Madrid2019, particularly in capturing the phase of the signal in the *Q* > 6 Å^–1^ region in both 3 and 6 m solutions. Neither model fully captures the low-*Q* region for the 6 m solution, which reflects long-range structural information in *r*-space.

**Figure 5:**
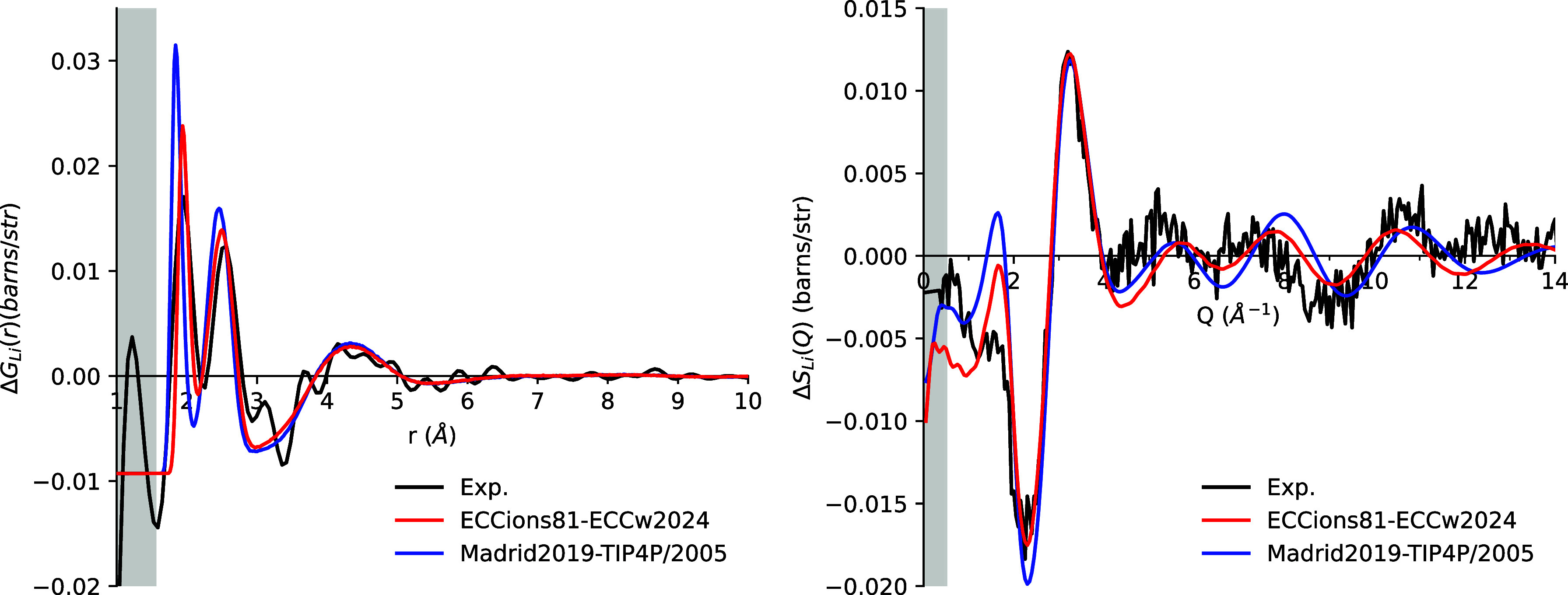
First-order difference functions Δ*G*_Li_(*r*) (left) and Δ*S*_Li_(*Q*) (right) for simulations of 3 m LiCl solution using the ECCions81 model with the ECCw2024 water model (red), the Madrid2019 ion model with the TIP4P/2005 water model (blue), and experimental data (black). Gray shading indicates regions where the experimental signal consists of only noise (in *r*-space) or falls outside the measurement range (in *Q*-space).

#### Sodium

For sodium, in order to emphasize the correlations between ions and water, the signal of 4 m NaCl, *G*_Na_(*r*), was subtracted from that of 4 m KCl, *G*_K_(*r*), or 4 m LiCl, *G*_Li_(*r*):

10 $$\begin{array}{c} \Delta G_{\mathrm{NaK}}\left(r\right)=G_{K}\left(r\right)-G_{\mathrm{Na}}\left(r\right)\\ \Delta G_{\mathrm{NaLi}}\left(r\right)=G_{\mathrm{Na}}\left(r\right)-G_{\mathrm{Li}}\left(r\right) \end{array}$$

Figure 6 presents a comparison between simulations and neutron diffraction results for Δ*G*_NaK_(*r*). In the corresponding *Q*-space data (Figure S10), a drop in the experimental baseline at 6 Å^–1^ is observed due to the absence of 4 m KCl data beyond this point. Despite this, the available experimental data are sufficient to resolve the primary peaks, whose positions and heights are retained after Fourier transformation into *r*-space. In *Q*-space, both ECCions81 and Madrid2019 show phase differences relative to the experiment but successfully capture the positions and intensities of the first two major peaks. In *r*-space, both models reproduce the main minima and maxima of the experimental signal. ECCions81 predicts a shallower Na–O minimum at 2.3 Å than Madrid2019, which is in better agreement with the experiment. A broader first peak—arising from K–O and O–O correlations—is also observed in ECCions81 compared to the experimental data (for further discussion, see Supporting Information).

**Figure 6:**
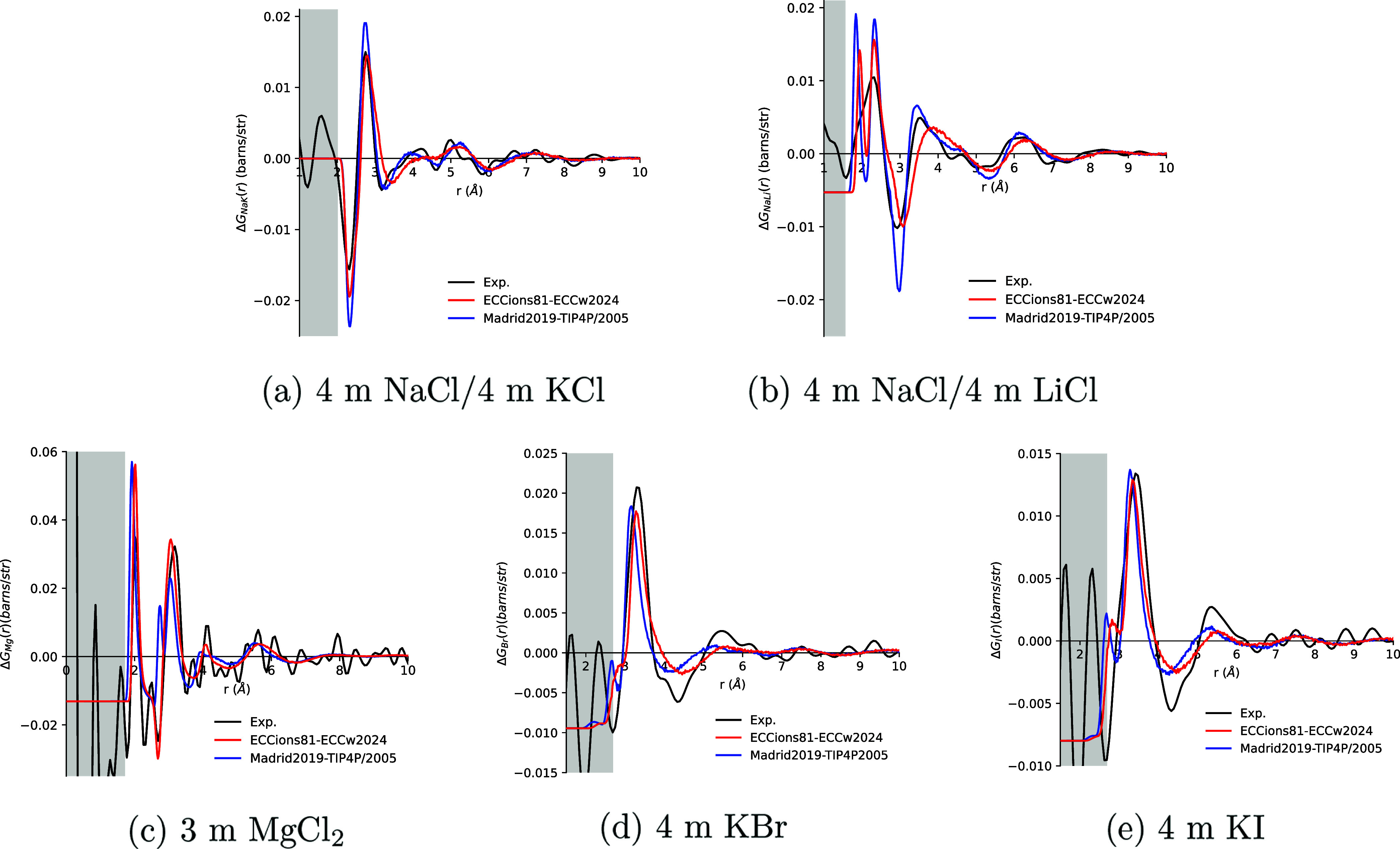
First-order difference functions, Δ*G*_MW_(*r*), for simulations of various cations or anion “M” in solutions using the ECCions81 model with ECCw2024 water (red), the Madrid2019 ion model with TIP4P/2005 water (blue), and experimental data (black). Panels (a) and (b) show Δ*G*_MW_(*r*) for Na^+^ in 4 m NaCl, subtracted by that of (a) K^+^ in 4 m KCl and (b) Li^+^ in 4 m LiCl. Panels (c)–(e) show Δ*G*_MW_(*r*) for (c) Mg^2+^ in 3 m MgCl_2_, (d) Br^–^ in 4 m KBr, and (e) I^–^ in 4 m KI.

Figure 6 also shows a comparison between the experimental Δ*G*_NaLi_(*r*) and results from the simulations. ECCions81 and Madrid2019 both exhibit two distinct peaks between 1.8 and 2.7 Å, while the experimental signal shows a single merged peak in this region. ECCions81 more accurately reproduces the experimental peak heights. Beyond 3 Å, however, ECCions81 shows a significant phase shift, whereas Madrid2019 aligns better with the experimental features. In *Q*-space, a similar trend is observed (Figure S12): ECCions81 deviates from the experiment above 4 Å^–1^, while Madrid2019 maintains consistent agreement across the full range. These discrepancies may partly result from inaccuracies in the O–O correlations of the LiCl reference system, which are not fully captured in the NDIS data (for further discussion, see Supporting Information).

#### Magnesium

The hydration structures of Mg^2+^ in a solution of 3 m MgCl_2_ obtained from simulations using the ECCions81 and Madrid2019 models, compared with neutron diffraction data, are shown in Figure 6. To emphasize ion–water correlations, the total signal was adjusted by the O–O correlation in pure ″null″ water.

The ECCions81 model demonstrates better agreement with the experimental signal than the Madrid2019 model. Specifically, the ECCions81 model accurately reproduces the position of the first peak at 2 Å corresponding to the correlation between Mg and O (Figure S15), although the peak height is slightly overestimated compared with the experimental data. In contrast, the Madrid2019 model shows a significantly higher peak with a 0.1 Å deviation in the peak position. Neither model captures the first minimum at approximately 2.3 Å. Additionally, the ECCions81 model accurately reproduces the second minimum at 2.6 Å, which corresponds to O–O correlations, while the Madrid2019 model displays a high peak at this position. This indicates that the ECCions81 model more accurately captures the influence of Mg^2+^ and Cl^–^ ions on the O–O interactions. For the second main peak at around 3 Å, the ECCions81 model matches both the height and position of the experimental data well, whereas the Madrid2019 model shows a slight deviation in position and a significantly lower peak height. In *Q*-space, the ECCions81 model also exhibits better agreement with the experimental signal compared to the Madrid2019 model. This is particularly evident at the first peak at 2.4 Å^–1^ and in the range between 6 and 10 Å^–1^, where the ECCions81 model demonstrates better phase matching. Finally, convergence data concerning ion pairing are presented in Supporting Information.

#### Chloride

Chloride is the anion present in all of the solutions discussed above. This makes the Cl–water interaction a critical anchor point in developing the ECCions81 model. While detailed structural data are only available from NDIS measurements on a 6 m LiCl solution, this data set serves as a valuable benchmark for directly optimizing the Cl–water interaction.

Figure 7 compares the simulation results of the first-order difference functions in the *r*-space and *Q*-space for Cl^–^ with NDIS data. While *r*-space and *Q*-space representations contain, in principle, the same information, they emphasize different aspects. Namely, *Q*-space highlights peak positions related to phasing, whereas *r*-space facilitates the interpretation of peak heights.

**Figure 7:**
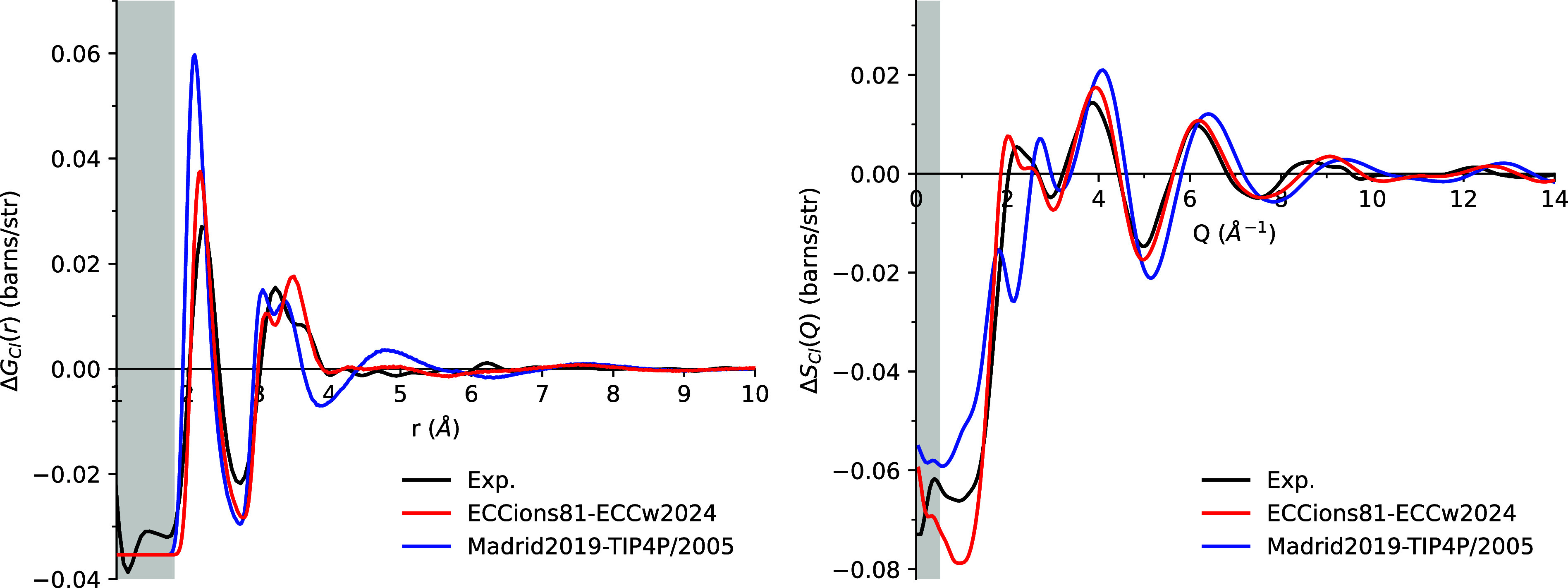
First-order difference functions Δ*G*_Cl_(*r*) (left) and Δ*S*_Cl_(*Q*) (right) for simulations of 6 m LiCl solution using the ECCions81 model with the ECCw2024 water model (red), the Madrid2019 ion model with the TIP4P/2005 water model (blue), and experimental data (black). Gray shading indicates regions where the experimental signal consists of only noise (in *r*-space) or falls outside the measurement range (in *Q*-space).

In *r*-space, both ECCions81 and Madrid2019 models capture the first and second peaks, corresponding to the first and second solvation shells, which are clearly separated by a deep minimum at 2.8 Å. However, the Madrid2019 model significantly overestimates the height of the first peak, representing the correlations between Cl^–^ and hydrogen in water (Figure S6b). Additionally, the phases of the first and second peaks in Madrid2019 begin and end at shorter distances than those observed experimentally, indicating a tighter hydration shell for Cl^–^ compared to experiment.

In contrast, ECCions81 shows a smaller first peak, partly due to the presence of Li–Cl contact ion pairs (CIPs) within the first hydration shell (Figure S6a), with a CIP value of 0.43 (Table 2). This pairing reduces the water density around Cl^–^ in the first hydration shell. In Madrid2019, such pairing is deliberately avoided, resulting in a CIP value of zero. The ECC model also better matches the phase of the second peak than Madrid2019, which includes contributions from the first peaks of *g*_ClO_ and the second peak of *g*_ClH_. Both ECCions81 and Madrid2019 models fail to fully capture the second peak’s height and position, while also displaying a deeper minimum between the first and second peaks. The Madrid2019 model achieves a comparable peak height but exhibits a narrower width and a shorter starting position, deviating from the experimental observations. Conversely, ECCions81 accurately reproduces the position of the second peak but underestimates its height.

In *Q*-space, the Madrid2019 model fails to accurately capture the first peak between 2 and 3 Å^–1^, where a single peak splits into two distinct peaks. In contrast, ECCions81 successfully reproduces the position of the first peak and provides a better match for peaks at higher *Q* values, with only a phase mismatch observed around 8 Å^–1^. In the low *Q* region (<2 Å^–1^), neither model captures the signal effectively, which corresponds to long-range hydration structures that are hard to observe in the *r*-space signal at distances greater than 4 Å.

#### Bromide

Similar to MgCl_2_, the neutron diffraction data for KBr were processed by subtracting a weighted signal of the O–O correlations in pure ″null″ water. The resulting data, along with those obtained from MD simulations using the ECCions81 and Madrid2019 ion models, are shown in Figure 6. In *r*-space, ECCions81 exhibits overall better agreement with the experimental signal, capturing the phase of peaks and valleys more accurately. In contrast, Madrid2019 predicts a shorter Br–O distance in the first hydration shell (Figure S20). However, both models fail to fully reproduce the amplitudes of all the peaks and valleys.

The most notable difference between ECCions81 and Madrid2019 is the peak at 2.8 Å predicted by Madrid2019, which is absent in the experimental data and appears only as a small bump in the ECCions81 signal, indicating that ECCions81 provides a structure closer to the experimental one. As shown in Figure S20, this peak or bump originates from K–O correlations, which are partially canceled by differences in O–O correlations between pure water and the KBr solution (Δ*G*_OO_). As discussed in sections Potassium and Chloride, Madrid2019 overestimates K–O correlations. Here, it also exhibits a larger Δ*G*_OO_ compared to ECCions81, though these differences are insufficient to fully compensate for the overestimated K–O signal, leaving a noticeable peak at this position (for further details and discussion of *Q*-space data, see Supporting Information).

#### Iodide

The results for the KI solutions are similar to those for KBr. As shown in Figure 6, Madrid2019 again exhibits a peak at 2.8 Å in *r*-space, originating from K–O correlations, which are partially canceled by Δ*G*_OO_ (Figure S22). ECCions81, in contrast, shows a smaller bump at 2.9 Å from the same source and achieves a better phase match for the second peak. While both models capture the height of the first peak, neither accurately reproduces the width of the first peak, the amplitude of the first valley at 4.3 Å, or the second peak at 5.3 Å (for further details and discussion of *Q*-space data, see Supporting Information).

### Transferability

As emphasized above, in parallel with structural neutron diffraction data, the ECCions81 model was calibrated to also reproduce macroscopic physical properties, such as viscosity and density, in other chloride-containing solutions, ensuring broader transferability and consistency across diverse ionic environments.

The transferability of ECCions81 across water models was further tested by using TIP4P/2005. Despite having significantly different dielectric constants, ECCw2024 and TIP4P2005 exhibit similar behavior for both density and viscosity (Figure S23). Consequently, the differences in our ion model performance between these two water frameworks are minimal with deviations smaller than 1% (Tables S5 and S6). The only exception is the water self-diffusion coefficient, where neat water simulations with ECCw2024 yield *D*_OW_ values approximately 4% lower than those with TIP4P/2005.^12^ This difference propagates to electrolyte solutions at low concentrations (<1 m), where *D*_OW_ values calculated with ECCw2024 remain 5% lower than those obtained with TIP4P/2005, as shown in Table S7. At higher concentrations (>3 m), nevertheless, *D*_OW_ values converge to similar values for both water models.

Similarly, the choice of the water model, among good ones, has only a minor influence on the RDFs for all investigated cases. As an example of such a small effect, switching from ECCw2024 to TIP4P/2005 results in a slightly smaller second peak in the *r*-space data for Ca^2+^, corresponding to the Ca–H correlation (Figure 8). In *Q*-space, the difference is barely noticeable in the 4–8 Å^–1^ range.

**Figure 8:**
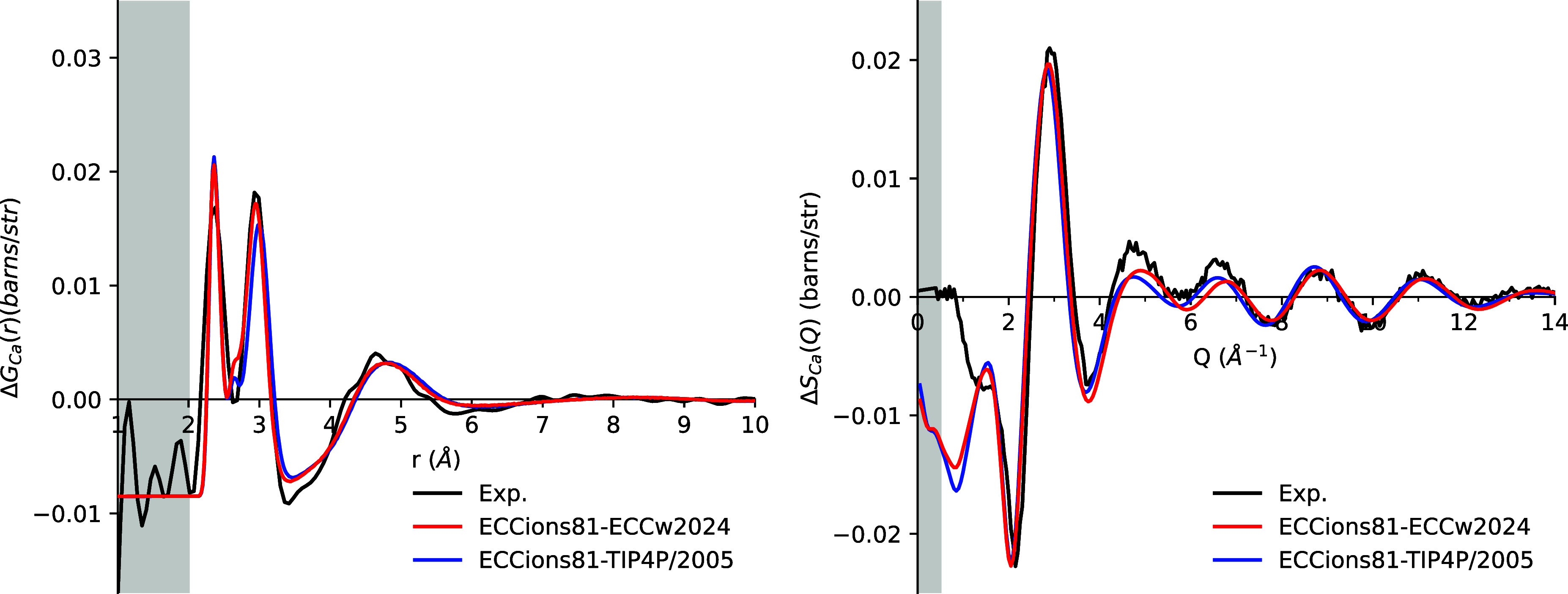
First-order difference functions Δ*G*_Ca_(*r*) (left) and Δ*S*_Ca_(*Q*) (right) for simulations of 4 m CaCl_2_ solution using the ECCions81 model with the ECCw2024 water model (red), the TIP4P/2005 water model (blue), and experimental data (black).

## Conclusion

In this study, we developed a charge-scaled ion force field, consistent with the a priori ECC-compatible ECCw2024 water model, for biologically relevant cations (Li^+^, Na^+^, K^+^, Mg^2+^, and Ca^2+^) and anions (Cl^–^, Br^–^, and I^–^) in aqueous solutions. The optimization process was conducted through an automated framework incorporating an LGP model to achieve global optimization across multiple target properties, including experimental neutron diffraction data, solution densities, and viscosities. The resulting ECCions81 parametrization was further validated against experimental data, where it compared favorably with, and in many aspects outperforming, the hitherto best ion model, i.e., Madrid2019, which is also a charge-scaled model.

The ECCions81 models, employing a scale factor of 0.81, demonstrated excellent agreement with experimental ion hydration structures across a wide range of concentrations. In particular, emphasizing correct cation–anion pairing significantly improved the ability of the model to reproduce neutron scattering data, in particular, the positions and heights of key peaks in the *r*-space for Li^+^, Na^+^, K^+^, Cl^–^, Mg^2+^, and Ca^2+^ ions. This performance surpassed that of the Madrid2019 model, which exhibits minimal ion pairing in the first hydration shell except for K^+^.

In terms of thermodynamic properties, the ECCions81 model successfully captures the concentration dependence of solution densities, showing comparable accuracy to Madrid2019, with the exception of CaCl_2_, where the maximum deviation nevertheless did not exceed 4%. The ECCions81 model also exhibits high accuracy for viscosity and water self-diffusion coefficients, maintaining deviations typically below 10% even at high concentrations (up to 4 m). In comparison, the Madrid2019 model displays significant deviations from experimental values at higher concentrations, particularly for viscosity and diffusion coefficients, where errors reached 60% in some cases.

Nevertheless, certain limitations of the ECCions81 model have been identified. Variations in the first-order difference function between 4 m NaCl and 4 m LiCl suggest that, while the ECC Li^+^ model accurately captures ion–solvent correlations, it fails to accurately reproduce changes in O–O correlations induced by Li^+^. Additionally, a preliminary calculation of Na^+^ solubility using the method developed by the van der Vegt group^45^ yielded a value of only about 1 m, significantly lower than the experimental value of 6.1 m. This discrepancy points to potentially overly strong ion pairing in the first hydration shell, possibly due to overfitting to the NDIS data. This issue may be partially addressed by adopting a less steep short-range potential, such as a Buckingham potential or a machine learning potential. These potentials should produce more realistic structural features without relying on artificially strong ion pairing. The resulting RDFs from such improved potentials may then serve as more suitable references for reoptimizing the Lennard-Jones parameters. Until then, using the Madrid2019 force field remains the best option for highly concentrated ionic solutions.^46^

Finally, note that the ionic scaling factor fully consistent with ECC is approximately 0.75 for a water model with a dielectric constant around 45. Nevertheless, we have previously shown that dielectric screening is slightly attenuated for ions in close contact,^28^ which is relevant when the parametrization process considers ion pairing. Still, there is a small mismatch between the optimal scaling factor obtained here and the dielectric constant of the ECCw2024 water model. Namely, an ionic charge scaling of 0.81 combined with a water model with a dielectric constant of 45 corresponds to an effective dielectric constant of around 70, which is slightly lower than the experimental value of 78. This motivates a future quest for a fully consistent combination of the ionic scaling factor and the dielectric constant of the water model. While our study shows robust behavior of water and ion models across a relatively broad range of dielectric constants and scaling factors, preliminary results, which form the basis for our future work, point toward a potential sweet spot with scaling of ionic charges by ∼0.8 in water with a dielectric constant of about 50–55.

## Supplementary Material



## Notes

Detailed methods for comparing simulation RDFs with neutron diffraction results. Results on densities, viscosities, and water diffusion coefficients of electrolyte solutions modeled with ECCions81 and Madrid2019. Additional results of weighted-component comparisons of simulation RDFs with neutron diffraction. Additional discussions on ECCions81 potassium, sodium, magnesium, bromide, and iodide ion models. Results on the transferability of ECCions81 ion models (PDF)

## References

[1] Leontyev I. V., Stuchebrukhov A. A. (2010). Electronic Continuum Model for Molecular Dynamics Simulations
of Biological Molecules. J. Chem. Theory Comput..

[2] Leontyev I., Stuchebrukhov A. (2011). Accounting
for electronic polarization in non-polarizable
force fields. Phys. Chem. Chem. Phys..

[3] Kirby B. J., Jungwirth P. (2019). Charge Scaling
Manifesto: A Way of Reconciling the
Inherently Macroscopic and Microscopic Natures of Molecular Simulations. J. Phys. Chem. Lett..

[4] Duboué-Dijon E., Javanainen M., Delcroix P., Jungwirth P., Martinez-Seara H. (2020). A practical guide to biologically relevant molecular
simulations with charge scaling for electronic polarization. J. Chem. Phys..

[5] Kohagen M., Mason P. E., Jungwirth P. (2014). Accurate Description
of Calcium Solvation
in Concentrated Aqueous Solutions. J. Phys.
Chem. B.

[6] Mason P. E., Ansell S., Neilson G. W., Rempe S. B. (2015). Neutron Scattering
Studies of the Hydration Structure of Li+. J.
Phys. Chem. B.

[7] Duboué-Dijon E., Mason P. E., Fischer H. E., Jungwirth P. (2018). Hydration
and Ion Pairing in Aqueous Mg^2+^ and Zn^2^ Solutions:
Force-Field Description Aided by Neutron Scattering Experiments and
Ab Initio Molecular Dynamics Simulations. J.
Phys. Chem. B.

[8] Martinek T., Duboué-Dijon E., Timr Š., Mason P. E., Baxová K., Fischer H. E., Schmidt B., Pluhařová E., Jungwirth P. (2018). Calcium ions in aqueous solutions: Accurate force field
description aided by ab initio molecular dynamics and neutron scattering. J. Chem. Phys..

[9] Berendsen, H. J. C. ; Postma, J. P. M. ; van Gunsteren, W. F. ; Hermans, J. Intermolecular Forces: Proceedings of the Fourteenth Jerusalem Symposium on Quantum Chemistry and Biochemistry Held in Jerusalem, Israel, 13–16, 1981; Pullman, B. , Ed.; Springer: Netherlands, pp. 331–342.

[10] Jorgensen W. L., Chandrasekhar J., Madura J. D., Impey R. W., Klein M. L. (1983). Comparison
of simple potential functions for simulating liquid water. J. Chem. Phys..

[11] Abascal J. L. F., Vega C. (2005). A general purpose model for the condensed phases of
water: TIP4P/2005. J. Chem. Phys..

[12] Cruces
Chamorro V., Jungwirth P., Martinez-Seara H. (2024). Building Water
Models Compatible with Charge Scaling Molecular Dynamics. J. Phys. Chem. Lett..

[13] Zeron I. M., Abascal J. L. F., Vega C. (2019). A force field of Li^+^,
Na^+^, K^+^, Mg^2+^, Ca^2+^, Cl^–^, and in aqueous solution based on the TIP4P/2005 water
model and scaled charges for the ions. J. Chem.
Phys..

[14] Nencini R., Tempra C., Biriukov D., Riopedre-Fernandez M., Cruces Chamorro V., Polák J., Mason P. E., Ondo D., Heyda J., Ollila O. H. S., Jungwirth P. (2024). Effective Inclusion of Electronic Polarization
Improves the Description
of Electrostatic Interactions: The prosECCo75 Biomolecular Force Field. J. Chem. Theory Comput..

[15] Shanks B. L., Sullivan H. W., Shazed A. R., Hoepfner M. P. (2024). Accelerated
Bayesian
Inference for Molecular Simulations using Local Gaussian Process Surrogate
Models. J. Chem. Theory Comput..

[16] Pluhařová E., Fischer H. E., Mason P. E., Jungwirth P. (2014). Hydration
of the chloride ion in concentrated aqueous solutions using neutron
scattering and molecular dynamics. Mol. Phys..

[17] Sindt J. O., Alexander A. J., Camp P. J. (2014). Structure and Dynamics of Potassium
Chloride in Aqueous Solution. J. Phys. Chem.
B.

[18] Badyal Y. S., Barnes A. C., Cuello G. J., Simonson J. M. (2004). Understanding the
Effects of Concentration on the Solvation Structure of Ca2+ in Aqueous
Solution. II: Insights into Longer Range Order from Neutron Diffraction
Isotope Substitution. J. Phys. Chem. A.

[19] Kohagen M., Mason P. E., Jungwirth P. (2016). Accounting
for Electronic Polarization
Effects in Aqueous Sodium Chloride via Molecular Dynamics Aided by
Neutron Scattering. J. Phys. Chem. B.

[20] Mason P. E., Ansell S., Neilson G. W. (2006). Neutron
diffraction studies of electrolytes
in null water: A direct determination of the first hydration zone
of ions. J. Phys.: Condens. Matter.

[21] Abraham M. J., Murtola T., Schulz R., Páll S., Smith J. C., Hess B., Lindahl E. (2015). GROMACS: High
performance
molecular simulations through multi-level parallelism from laptops
to supercomputers. SoftwareX.

[22] Hoover W. G. (1985). Canonical
dynamics: Equilibrium phase-space distributions. Phys. Rev. A.

[23] Parrinello M., Rahman A. (1981). Polymorphic transitions in single crystals: A new molecular
dynamics method. J. Appl. Phys..

[24] Essmann U., Perera L., Berkowitz M. L., Darden T., Lee H., Pedersen L. G. (1995). A smooth particle
mesh Ewald method. J. Chem. Phys..

[25] Hess B. (2002). Determining
the shear viscosity of model liquids from molecular dynamics simulations. J. Chem. Phys..

[26] Maginn E. J., Messerly R. A., Carlson D. J., Roe D. R., Elliot J. R. (2019). Best Practices
for Computing Transport Properties 1. Self-Diffusivity and Viscosity
from Equilibrium Molecular Dynamics Article v1.0. Living J. Comput. Mol. Sci..

[27] Yeh I.-C., Hummer G. (2004). System-Size Dependence of Diffusion
Coefficients and
Viscosities from Molecular Dynamics Simulations with Periodic Boundary
Conditions. J. Phys. Chem. B.

[28] Kostal V., Jungwirth P., Martinez-Seara H. (2023). Nonaqueous Ion Pairing Exemplifies
the Case for Including Electronic Polarization in Molecular Dynamics
Simulations. J. Phys. Chem. Lett..

[29] Hui C., de Vries R., Kopec W., de Groot B. L. (2025). Effective polarization
in potassium channel simulations: Ion conductance, occupancy, voltage
response, and selectivity. Proc. Natl. Acad.
Sci. U. S. A..

[30] Dočkal J., Lísal M., Moučka F. (2022). Polarizable force fields for accurate
molecular simulations of aqueous solutions of electrolytes, crystalline
salts, and solubility: Li+, Na+, K+, Rb+, F-, Cl-, Br-, I-. J. Mol. Liq..

[31] Dočkal J., Mimrová P., Lísal M., Moučka F. (2024). Structure
of aqueous alkali metal halide electrolyte solutions from molecular
simulations of phase-transferable polarizable models. J. Mol. Liq..

[32] Ikeda T., Boero M., Terakura K. (2007). Hydration
of alkali ions from first
principles molecular dynamics revisited. J.
Chem. Phys..

[33] Hofer T. S. (2022). Solvation
Structure and Ion-Solvent Hydrogen Bonding of Hydrated Fluoride, Chloride
and Bromide-A Comparative QM/MM MD Simulation Study. Liquids.

[34] Tongraar A., Hannongbua S., Rode B. M. (2010). QM/MM MD Simulations of Iodide Ion
(I-) in Aqueous Solution: A Delicate Balance between Ion-Water and
Water-Water H-Bond Interactions. J. Phys. Chem.
A.

[35] Azam S. S., Hofer T. S., Randolf B. R., Rode B. M. (2009). Hydration of Sodium­(I)
and Potassium­(I) Revisited: A Comparative QM/MM and QMCF MD Simulation
Study of Weakly Hydrated Ions. J. Phys. Chem.
A.

[36] Guàrdia E., Skarmoutsos I., Masia M. (2009). On Ion and Molecular Polarization
of Halides in Water. J. Chem. Theory Comput..

[37] Marcus Y. (2009). Effect of
Ions on the Structure of Water: Structure Making and Breaking. Chem. Rev..

[38] Ohtaki H., Radnai T. (1993). Structure and dynamics of hydrated ions. Chem. Rev..

[39] Ramos S., Barnes A. C., Neilson G. W., Capitan M. J. (2000). Anomalous X-ray
diffraction studies of hydration effects in concentrated aqueous electrolyte
solutions. Chem. Phys..

[40] Fulton J. L., Pfund D. M., Wallen S. L., Newville M., Stern E. A., Ma Y. (1996). Rubidium ion hydration
in ambient and supercritical water. J. Chem.
Phys..

[41] Laliberté M., Cooper W. E. (2004). Model for Calculating the Density of Aqueous Electrolyte
Solutions. J. Chem. Eng. Data.

[42] Sedano L. F., Blazquez S., Noya E. G., Vega C., Troncoso J. (2022). Maximum in
density of electrolyte solutions: Learning about ion-water interactions
and testing the Madrid-2019 force field. J.
Chem. Phys..

[43] Gámez F., Sedano L. F., Blazquez S., Troncoso J., Vega C. (2023). Building a
Hofmeister-like series for the maximum in density temperature of aqueous
electrolyte solutions. J. Mol. Liq..

[44] Blazquez S., Conde M. M., Vega C. (2023). Scaled charges for
ions: An improvement
but not the final word for modeling electrolytes in water. J. Chem. Phys..

[45] Chattopadhyay A., Mandalaparthy V., van der Vegt N. F. A. (2025). Determination of aqueous solubility
of NaCl in molecular dynamics simulation using the Kirkwood-Buff method. J. Chem. Phys..

[46] Blazquez S., Conde M. M., Abascal J. L. F., Vega C. (2022). The Madrid-2019 force
field for electrolytes in water using TIP4P/2005 and scaled charges:
Extension to the ions F-, Br-, I-, Rb+, and Cs. J. Chem. Phys..

