# Supplementary material for: Charge Scaling Force Field for Biologically Relevant Ions Utilizing a Global Optimization Method
Source: J Chem Theory Comput. 2025 Sep 4;21(18):9023–34. doi: 10.1021/acs.jctc.5c00873 (PMC12461915; doi:10.1021/acs.jctc.5c00873)
Supplement: Supplementary file 1 [file ct5c00873_si_001.pdf]

Supporting Information: Charge Scaling Force Field for Biologically Relevant Ions Utilizing a Global Optimization Method

Shujie Fan, Philip E. Mason, Victor Cruces Chamorro, Brennon Shanks, Hector Martinez-Seara,* and Pavel Jungwirth*

Institute of Organic Chemistry and Biochemistry, Academy of Sciences of the Czech Republic, Flemingovo nam. 2, CZ-16610 Prague 6, Czech Republic

E-mail: hseara@gmail.com; pavel.jungwirth@uochb.cas.cz

Contents

1 Comparison between simulation RDFs and neutron diffracton results S-3

2 Parameters of ion models S-5

3 Physical properties for best optimized models S-6

4 Hydration structure S-12

4.1 Lithium S-12

4.2 Chloride S-13

4.3 Calcium S-14

4.4 Potassium S-14

4.5 Sodium S-16

4.6 Magnesium S-20

4.7 Bromide S-25

4.8 Iodide S-27

5 Water effect in physical and structural properties for ECCions81 S-28

References S-33

1 Comparison between simulation RDFs and neutron diffracton results

For solutions of salts (M^+Cl^-) measured using NDIS in heavy water (D_2O), the RDFs were compared with first order difference functions through a weighted summation:

$$\Delta G_{\mathrm{M}}(r) = K_{\mathrm{MO}} g_{\mathrm{MO}}(r) + K_{\mathrm{MD}} g_{\mathrm{MD}}(r) + K_{\mathrm{MCl}} g_{\mathrm{MCl}}(r) + K_{\mathrm{MM}} g_{\mathrm{MM}}(r) - K \tag{1}$$

where the prefactors are defined as:

$$\begin{aligned} K_{\mathrm{M}\alpha} &= 2c_{\mathrm{M}} c_\alpha \bar{b}_\alpha \Delta \bar{b}_{\mathrm{M}} \\ K_{\mathrm{MM}} &= c_{\mathrm{M}}{}^2 \left(\bar{b}_{\mathrm{M}}{}^2 - \bar{b}_{\mathrm{M'}}{}^2 \right) \\ K &= K_{\mathrm{MO}} + K_{\mathrm{MD}} + K_{\mathrm{MCl}} + K_{\mathrm{MM}} \end{aligned} \tag{2}$$

Here, c_α represents the atomic concentration of species α, $\bar{b}\alpha$ is its mean coherent neutron scattering length, and $\Delta\bar{b}_{\mathrm{M}} = \bar{b}_{\mathrm{M}} - \bar{b}_{\mathrm{M'}}$ is the difference in scattering lengths between M and its isotope M′. Note that while the experiments were conducted in D_2O, the simulations employed normal water (H_2O) instead. For Cl^-, which was measured in 6 m LiCl solution, a similar weighted summation was applied:

$$\Delta G_{\mathrm{Cl}}(r) = K_{\mathrm{ClO}} g_{\mathrm{ClO}}(r) + K_{\mathrm{ClD}} g_{\mathrm{ClD}}(r) + K_{\mathrm{ClLi}} g_{\mathrm{ClLi}}(r) + K_{\mathrm{ClCl}} g_{\mathrm{ClCl}}(r) - K \tag{3}$$

For solutions of salts measured in "null" water, involving M and its counterion X, where M could be a cation or an anion, the RDFs were combined into a total signal to compare with experimental data:

$$G_{\mathrm{M}}(r) = K_{\mathrm{OO}} g_{\mathrm{OO}}(r) + K_{\mathrm{MO}} g_{\mathrm{MO}}(r) + K_{\mathrm{XO}} g_{\mathrm{XO}}(r) + K_{\mathrm{MM}} g_{\mathrm{MM}}(r) + K_{\mathrm{XX}} g_{\mathrm{XX}}(r) + K_{\mathrm{MX}} g_{\mathrm{MX}}(r) - K \tag{4}$$

where the prefactors are defined as:

$$K_{\alpha\beta} = 2c_\alpha c_\beta \bar{b}_\beta \bar{b}_\alpha$$
$$K = K_{\mathrm{OO}} + K_{\mathrm{MO}} + K_{\mathrm{XO}} + K_{\mathrm{MM}} + K_{\mathrm{XX}} + K_{\mathrm{MX}} \tag{5}$$

The prefactors, summarized in Table S2, indicate that approximately 80% of the total signal $G_{\mathrm{M}}(r)$ originates from $g_{\mathrm{OO}}(r)$, representing oxygen-oxygen correlations. To highlight the contributions from ion-water correlations, we adjusted the signals as follows:

1. For $MgCl_2$, KBR and KI, the oxygen-oxygen contribution from pure water was scaled and subtracted:
$$\Delta G_{\mathrm{MW}}(r) = G_{\mathrm{M}}(r) - K_{\mathrm{OO}}(g_{\mathrm{OO}}^{\mathrm{H_2O}}(r) - 1) \tag{6}$$
where $g_{\mathrm{OO}}^{\mathrm{H_2O}}(r)$ represents the oxygen-oxygen RDF from pure water.

2. For NaCl, the total $G_{\mathrm{M}}(r)$ was compared against signals from 4 m KCl or 4 m LiCl solutions:,
$$\Delta G_{\mathrm{KNa}}(r) = G_{\mathrm{K}}(r) - G_{\mathrm{Na}}(r)$$
$$\Delta G_{\mathrm{NaLi}}(r) = G_{\mathrm{Na}}(r) - G_{\mathrm{Li}}(r) \tag{7}$$

The RDFs can also be compared with structure factors $S(Q)$ which were directly measured in experiments by fourier transformation.

Table S1: Prefactors for solutions of ions measured using NDIS in units of barns per steradian (10^{-3} barns/str).

Conc.	4 m	4 m	6 m	6 m	3 m
M	K^+	Ca^{2+}	Li^+	Cl^-	Li^+
X	Cl^-	Cl^-	Cl^-	Li^+	Cl^-
K_{MO}	1.3417	2.409	4.7281	10.402	2.7389
K_{MD}	3.0850	5.424	10.8718	23.918	6.2978
K_{MX}	0.1594	0.578	0.8426	-0.368	0.2441
K_{MM}	0.0479	0.098	0.0044	1.426	-0.0028
K	4.6339	8.509	16.4469	35.378	9.2780

Table S2: Prefactors for solutions of ions measured in "null" water in units of barns per steradian (10^{-3} barns/str).

Conc.	4 m	4 m	4 m	4 m	4 m	3 m
M	Na^+	Li^+	K^+	Br^-	I^-	Mg^{2+}
X	Cl^-	Cl^-	Cl^-	K^+	K^+	Cl^-
K_{MM}	0.069	0.019	0.07	0.242	0.1446	0.08
K_{MX}	0.365	-0.191	2.592	0.264	0.2043	0.6
K_{MO}	3.070	-1.610	3.137	5.740	4.438	3.4
K_{XX}	0.481	0.481	0.481	0.07	0.07	1.10
K_{XO}	8.101	8.101	8.101	3.137	3.137	12.0
K_{OO}	34.091	34.091	34.091	34.091	34.091	33.7
K	46.177	40.894	48.47	43.55	42.09	50.9

2 Parameters of ion models

Table S3: Pairwise Lennard-Jones σ, ϵ parameters for ECCions81 model and Madrid2019 ion model[S1] in ECCw2024 water, including Li^+, Na^+, K^+, Mg^{2+}, Ca^{2+}, Cl^-, Br^-, and I^-. For ECCions81, the parameters for cation-oxygen and cation-anion interactions were calculated using the Lorentz-Berthelot combination rules. The last column reports the difference in σ values between the ECCions81 and Madrid2019 models.

	**ECCions81**		**Madrid2019**		
	σ (nm)	ϵ (kJ/mol)	σ (nm)	ϵ (kJ/mol)	$\Delta\sigma$ (nm)
Li-O	0.233524	0.406696	0.212000	0.700650	0.021524
Li-Cl	0.272530	0.394097	0.270000	1.282944	0.002530
Na-O	0.276384	0.343128	0.260838	0.793388	0.015546
Na-Cl	0.315390	0.332498	0.300512	1.438894	0.014878
K-O	0.306240	0.801757	0.289040	1.400430	0.017200
K-Cl	0.345246	0.776918	0.339700	1.400000	0.005546
K-Br	0.367901	0.981846	0.379879	0.425940	-0.011978
K-I	0.410709	1.003304	0.400550	0.536590	0.010159
Mg-O	0.219422	1.851638	0.181000	12.000000	0.038422
Mg-Cl	0.258428	1.794273	0.300000	3.000000	-0.041572
Ca-O	0.278123	0.828020	0.240000	7.250000	0.038123
Ca-Cl	0.317129	0.802367	0.315000	1.000000	0.002129
Cl-O*	0.352611	0.723797	0.423867	0.061983	-0.071256
Br-O*	0.360099	1.172294	0.419850	0.100000	-0.059751
I-O*	0.372802	0.921039	0.434950	0.100000	-0.062148

3 Physical properties for best optimized models

Table S4: Comparison of oxygen coordination number of cations and anions (HN^O), hydrogen coordination number of anions (HN_a^H), and ion–oxygen distances at 1 m for the ECCions81 force field, the DLM/2022-BK3 force field,[S2] hybrid QM/MM simulations,[S3–S7] and experimental data.[S8–S11]

**Ion**	**Model**	HN^O	HN_a^H	d_{ion-O_w} (Å)
Li^+	ECCions81	3.9		1.94
	DLM/2022	4.0		1.94
	QM/MM	4.0		1.99
	Exp.	4.0-4.5		1.90-2.25
Na^+	ECCions81	5.1		2.32
	DLM/2022	5.7-5.8		2.34
	QM/MM	5.1-6.5		2.33-2.40
	Exp.	4.0-8.0		2.42-2.50
K^+	ECCions81	6.5		2.78-2.80
	DLM/2022	6.8-7.0		2.73
	QM/MM	6.2-8.3		2.78-2.85
	Exp.	3.2-8.0		2.70-2.95
Cl^-	ECCions81	5.9-6.1	5.4-5.6	3.06-3.08
	DLM/2022	6.1-6.4	5.7-5.9	3.20
	QM/MM	5.5-8.1		3.11-3.25
	Exp.	6.0		2.91-3.14
Br^-	ECCions81	6.2	5.6	3.28
	DLM/2022	7.2-7.5	5.95-6.25	3.20
	QM/MM	5.5-8.1		3.31-3.33
	Exp.	6.0		3.26-3.35
I^-	ECCions81	6.1	5.7	3.36
	DLM/2022	6.8-13	5.9-6.3	3.69-3.70
	QM/MM	6.6-8.8	3.0-5.1	3.45-3.75
	Exp.	4.2-6.9		3.63-3.70

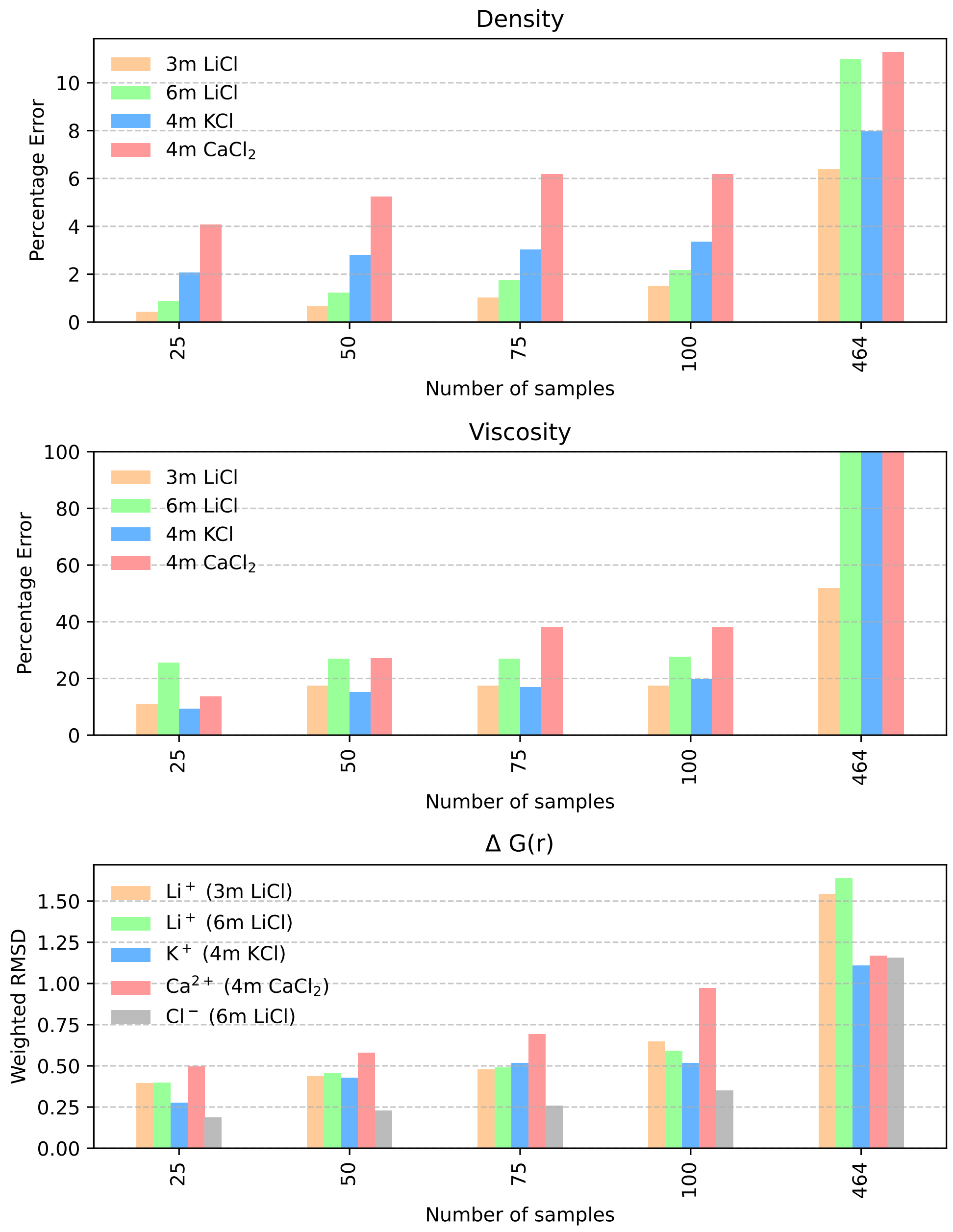

Figure S1: Maximum percentage error of density and viscosity, and the maximum weighted RMSD of the first-order difference, $\Delta G(r)$, between experimental data and predictions from the best-performing parameter sets. Results are shown for the top 25, 50, 75, 100, and all 464 samples from the optimization of Li^+, K^+, Ca^{2+}, and Cl^-. The RMSD of $\Delta G(r)$ was scaled by the total prefactor of each corresponding solution, as defined in Table S1. The upper limit for the viscosity error plot was set to 100% to facilitate comparison across samples; only the 464-sample case exceeded this threshold.

Table S5: Densities of ionic solutions predicted by simulations (kg/m^3). Values with deviations greater than 0.5% are highlighted in blue, and those exceeding 2% are highlighted in red.

Conc.	Salt	Exp.	ECCions81		Madrid2019	
			ECCw2024	TIP4P/2005	ECCw2024	TIP4P/2005
0.24	LiCl	1003.02	1003.53(20)	1002.21(9)	1003.09(20)	1002.59(16)
1.00	LiCl	1020.71	1021.03(13)	1019.77(27)	1020.28(11)	1018.70(7)
3.00	LiCl	1062.00	1062.31(9)	1059.90(16)	1061.34(23)	1059.57(12)
4.00	LiCl	1080.81	1080.85(13)	1078.49(9)	1080.28(8)	1078.10(19)
6.00	LiCl	1115.80	1115.51(12)	1112.86(8)	1116.62(15)	1114.56(16)
0.24	NaCl	1006.69	1007.64(14)	1006.86(17)	1007.61(26)	1007.06(28)
1.00	NaCl	1036.12	1037.87(9)	1037.04(13)	1037.32(28)	1036.34(15)
3.00	NaCl	1106.03	1108.66(7)	1107.34(12)	1106.99(17)	1105.92(20)
4.00	NaCl	1137.30	1140.15(14)	1138.85(12)	1138.02(15)	1137.49(17)
0.24	KCl	1008.07	1008.20(15)	1007.54(19)	1009.28(16)	1008.26(20)
1.00	KCl	1041.37	1039.98(19)	1038.98(13)	1042.56(13)	1041.91(7)
3.00	KCl	1118.58	1112.08(20)	1111.51(16)	1119.40(11)	1118.79(9)
4.00	KCl	1152.25	1143.53(27)	1143.18(17)	1152.59(13)	1152.50(19)
0.24	KBr	1017.01	1016.99(19)	1016.43(9)	1017.63(24)	1016.90(27)
1.00	KBr	1077.46	1075.70(12)	1074.46(17)	1078.42(20)	1077.45(14)
3.00	KBr	1218.68	1209.84(9)	1208.88(32)	1218.42(14)	1218.42(11)
4.00	KBr	1280.93	1267.88(22)	1266.94(13)	1280.19(8)	1280.56(19)
0.24	KI	1025.50	1026.42(13)	1026.02(16)	1026.18(13)	1025.84(9)
1.00	KI	1110.91	1113.02(13)	1112.20(18)	1112.12(13)	1111.28(22)
3.00	KI	1306.61	1310.07(22)	1310.07(22)	1309.54(6)	1309.82(24)
4.00	KI	1391.38	1396.08(21)	1395.72(35)	1395.07(21)	1396.17(17)
0.24	$MgCl_2$	1015.24	1016.50(31)	1015.00(15)	1015.85(6)	1015.06(16)
1.00	$MgCl_2$	1070.11	1070.68(7)	1068.31(13)	1069.90(19)	1067.90(12)
3.00	$MgCl_2$	1195.41	1196.23(21)	1193.06(28)	1195.45(27)	1192.38(16)
4.00	$MgCl_2$	1248.38	1250.69(43)	1246.71(31)	1250.44(35)	1248.95(69)
0.24	$CaCl_2$	1018.05	1017.54(31)	1016.29(14)	1019.89(16)	1018.76(10)
1.00	$CaCl_2$	1081.46	1074.75(9)	1072.08(18)	1084.98(19)	1082.20(15)
3.00	$CaCl_2$	1226.28	1194.23(38)	1189.31(26)	1226.02(40)	1221.93(68)
4.00	$CaCl_2$	1287.38	1241.87(35)	1236.93(14)	1282.89(48)	1279.28(53)

Table S6: Shear viscosities of ionic solutions predicted by simulations (mPa· s). Values with deviations greater than 10% are highlighted in blue, and those exceeding 25% are highlighted in red.

**Conc.**	**Salt**	**Exp.**	**ECCions81**		**Madrid2019**	
			**ECCw2024**	**TIP4P/2005**	**ECCw2024**	**TIP4P/2005**
0.24	LiCl	0.913	0.93(2)	0.90(4)	0.95(3)	0.92(2)
1.00	LiCl	1.01	1.07(2)	1.01(3)	1.10(5)	1.07(3)
3.00	LiCl	1.29	1.41(5)	1.40(6)	1.65(9)	1.64(6)
4.00	LiCl	1.46	1.66(5)	1.65(9)	2.03(11)	2.02(7)
6.00	LiCl	1.91	2.26(10)	2.24(11)	3.14(21)	3.22(30)
0.24	NaCl	0.91	0.93(2)	0.88(3)	0.96(4)	0.89(2)
1.00	NaCl	0.97	1.01(3)	0.96(4)	1.10(4)	1.02(3)
3.00	NaCl	1.20	1.22(3)	1.19(6)	1.49(5)	1.48(6)
4.00	NaCl	1.35	1.38(4)	1.35(5)	1.72(7)	1.76(7)
0.24	KCl	0.89	0.91(3)	0.87(3)	0.93(3)	0.88(2)
1.00	KCl	0.89	0.91(2)	0.89(2)	0.96(4)	0.95(2)
3.00	KCl	0.91	0.97(3)	0.93(3)	1.16(4)	1.12(2)
4.00	KCl	0.94	1.00(2)	1.00(3)	1.25(4)	1.23(3)
0.24	KBr	0.88	0.90(2)	0.84(3)	0.91(3)	0.88(3)
1.00	KBr	0.86	0.89(2)	0.85(3)	0.96(3)	0.92(2)
3.00	KBr	0.86	0.89(1)	0.86(2)	1.11(3)	1.07(2)
4.00	KBr	0.87	0.90(1)	0.88(2)	1.18(4)	1.17(2)
0.24	KI	0.87	0.91(3)	0.84(3)	0.91(3)	0.86(2)
1.00	KI	0.83	0.88(2)	0.85(3)	0.93(2)	0.90(2)
3.00	KI	0.81	0.84(2)	0.84(2)	1.03(3)	1.01(2)
4.00	KI	0.82	0.89(1)	0.87(2)	1.09(3)	1.09(2)
0.24	$MgCl_2$	0.97	0.99(3)	0.95(4)	1.00(4)	0.95(3)
1.00	$MgCl_2$	1.29	1.30(4)	1.25(6)	1.47(7)	1.39(4)
3.00	$MgCl_2$	2.96	2.87(14)	2.82(17)	4.17(36)	4.09(33)
4.00	$MgCl_2$	4.73	4.71(40)	4.65(30)	7.68(62)	7.64(77)
0.24	$CaCl_2$	0.91	0.97(2)	0.92(3)	1.00(4)	0.95(2)
1.00	$CaCl_2$	1.22	1.22(3)	1.15(3)	1.35(6)	1.30(4)
3.00	$CaCl_2$	2.20	2.23(10)	2.28(16)	3.05(21)	3.06(20)
4.00	$CaCl_2$	3.25	3.28(19)	3.18(20)	4.74(37)	4.84(40)

Table S7: Water self-diffusion coefficients of ionic solutions predicted by simulations (10^{-5} cm^2/s). Values with deviations greater than 10% are highlighted in blue, and those exceeding 25% are highlighted in red.

**Conc.**	**Salt**	**Exp.**	**ECCions81**		**Madrid2019**	
			**ECCw2024**	**TIP4P/2005**	**ECCw2024**	**TIP4P/2005**
0.24	LiCl	2.25	2.14(4)	2.25(4)	2.09(3)	2.16(4)
1.00	LiCl	2.09	1.89(4)	2.00(3)	1.81(3)	1.89(3)
3.00	LiCl	1.63	1.45(3)	1.48(3)	1.24(2)	1.28(2)
4.00	LiCl	1.44	1.27(2)	1.30(2)	1.02(2)	1.03(2)
6.00	LiCl	1.12	0.95(2)	0.96(1)	0.66(1)	0.67(1)
0.24	NaCl	2.27	2.12(3)	2.27(3)	2.10(4)	2.25(3)
1.00	NaCl	2.17	2.01(4)	2.11(4)	1.90(3)	2.00(4)
3.00	NaCl	1.87	1.73(4)	1.78(2)	1.46(3)	1.49(2)
4.00	NaCl	1.71	1.58(3)	1.62(2)	1.29(2)	1.28(2)
0.24	KCl	2.32	2.16(4)	2.31(3)	2.16(3)	2.26(3)
1.00	KCl	2.38	2.17(4)	2.28(4)	2.07(4)	2.14(4)
3.00	KCl	2.34	2.10(4)	2.18(4)	1.83(2)	1.89(3)
4.00	KCl	2.31	2.07(4)	2.11(5)	1.72(3)	1.75(3)
0.24	KBr	2.33	2.18(3)	2.35(4)	2.16(3)	2.29(3)
1.00	KBr	2.43	2.23(4)	2.36(3)	2.10(3)	2.20(4)
3.00	KBr	2.53	2.31(4)	2.37(4)	1.88(3)	1.94(4)
4.00	KBr	2.51	2.27(4)	2.34(4)	1.81(2)	1.85(3)
0.24	KI	2.36	2.15(3)	2.34(3)	2.15(3)	2.30(3)
1.00	KI	2.54	2.25(5)	2.34(4)	2.13(3)	2.23(4)
3.00	KI	2.66	2.38(3)	2.38(3)	2.00(3)	2.07(4)
4.00	KI	2.66	2.33(3)	2.36(4)	1.95(3)	1.96(3)
0.24	$MgCl_2$	2.12	2.02(4)	2.13(3)	2.00(3)	2.13(4)
1.00	$MgCl_2$	1.69	1.60(3)	1.68(3)	1.44(2)	1.53(2)
3.00	$MgCl_2$	0.86	0.80(2)	0.83(1)	0.57(1)	0.59(1)
4.00	$MgCl_2$	0.54	0.52(1)	0.53(1)	0.33(0)	0.34(0)
0.24	$CaCl_2$	2.20	2.06(4)	2.19(5)	2.01(3)	2.12(3)
1.00	$CaCl_2$	1.88	1.73(3)	1.81(4)	1.57(3)	1.64(3)
3.00	$CaCl_2$	1.07	1.04(2)	1.06(2)	0.77(1)	0.78(1)
4.00	$CaCl_2$	0.75	0.80(1)	0.80(1)	0.52(1)	0.52(1)

Table S8: Temperature of maximum density (TMD) and the density at the maximum for chloride solutions at 1 m and atmospheric pressure of ECCions81 and Madrid2019 models.[S12] Er% is the percentage deviation of the simulated from experimental results.[S12–S14]

		TMD (K)			ρ (kg/m^3)		
	Salt	Sim.	Exp.	Er%	Sim.	Exp.	Er%
ECCions81	LiCl	273.3	270.8	0.92	1024.71	1024.8	0.01
	NaCl	266.5	262.7	1.45	1044.17	1044.0	0.02
	KCl	269.7	265.0	1.77	1044.81	1047.5	0.26
	$MgCl_2$	267.2	261.3	2.25	1075.47	1076.5	0.10
	$CaCl_2$	264.0	253.6	4.11	1081.01	1092.4	1.04
Madrid2019	LiCl	270.7	270.8	0.04	1024.8	1024.8	0.17
	NaCl	260.7	262.7	0.76	1044.0	1044.0	0.01
	KCl	266.7	265.0	0.64	1047.9	1047.5	0.04
	$MgCl_2$	265.5	261.3	1.6	1073.5	1076.5	0.28
	$CaCl_2$	252.4	253.6	0.47	1092.2	1092.4	0.02

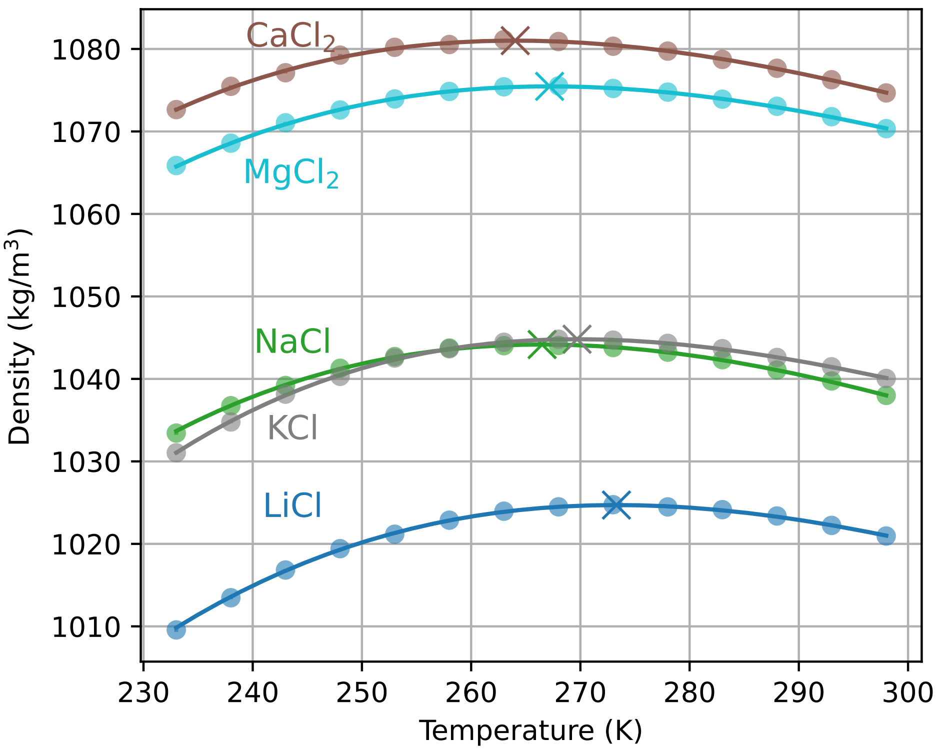

Figure S2: Density of various 1 m chloride solutions as a function of temperature at atmospheric pressure. Filled spheres represent simulation data points; solid lines correspond to cubic polynomial fits to the data. Crosses indicate the temperatures of maximum density (TMD).

4 Hydration structure

4.1 Lithium

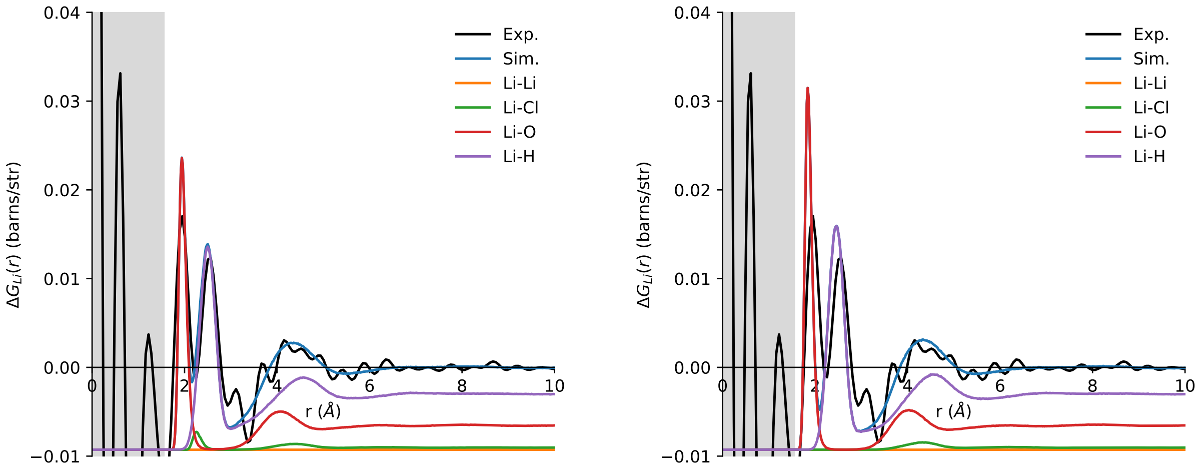

(a) ECCions81-ECCw2024

(b) Madrid2019-TIP4P/2005

Figure S3: First-order difference functions $\Delta G_{Li}(r)$ and the weighted components from simulations of 3 m LiCl solution using (a) the ECCions81 model the ECCw2024 water model, and (b) the Madrid2019 ion model with the TIP4P/2005 water model.

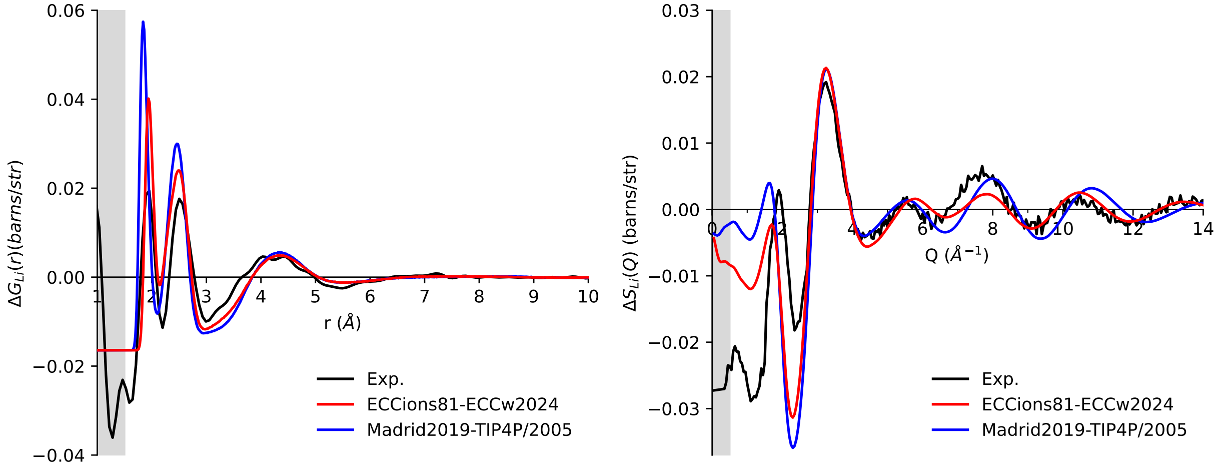

Figure S4: First-order difference functions $\Delta G_{Li}(r)$ (left) and $\Delta S_{Li}(Q)$ (right) for simulations of 6 m LiCl solution using the ECCions81 model with the ECCw2024 water model (red) and the Madrid2019 ion model with the TIP4P/2005 water model (blue), and experimental data (black). Gray shading indicates regions where the experimental signal consists of only noise (in r-space) or falls outside the measurement range (in Q-space).

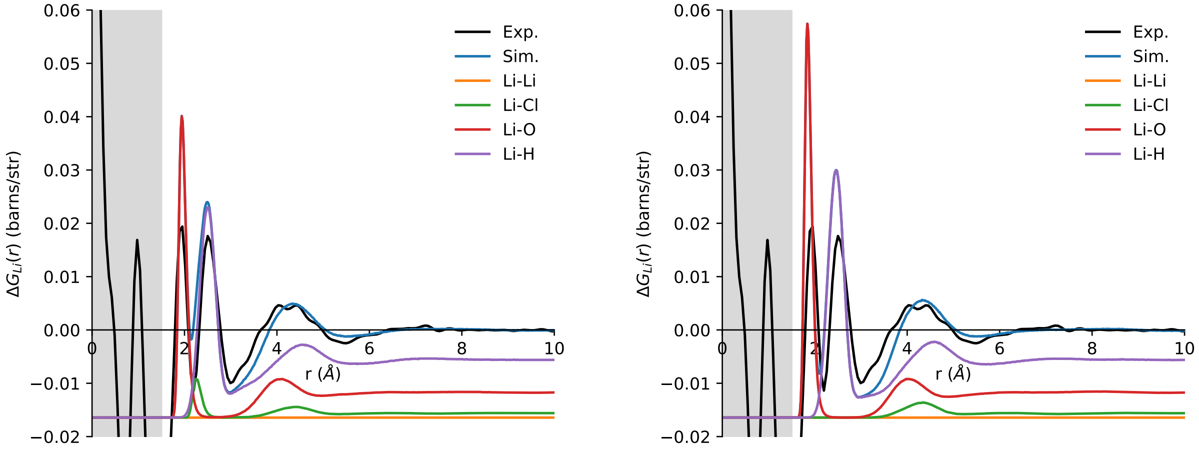

(a) ECCions81-ECCw2024

(b) Madrid2019-TIP4P/2005

Figure S5: First-order difference functions $\Delta G_{Li}(r)$ and the weighted components from simulations of the 6 m LiCl solution using (a) the ECCions81 model with the ECCw2024 water model and (b) the Madrid2019 ion model with the TIP4P/2005 water model.

4.2 Chloride

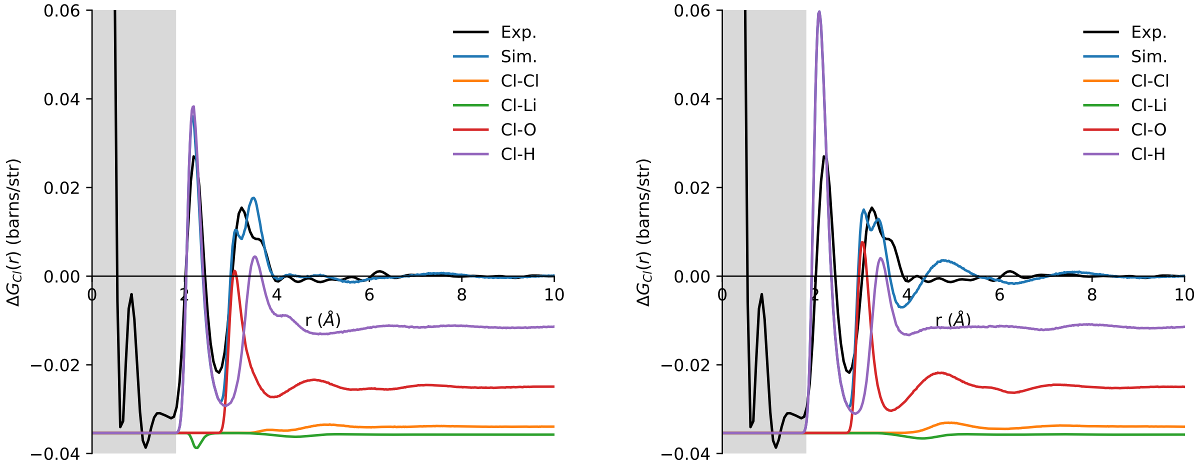

(a) ECCions81-ECCw2024

(b) Madrid2019-TIP4P/2005

Figure S6: First-order difference functions $\Delta G_{Cl}(r)$ and the weighted components from simulations of 6 m LiCl solution using (a) the ECCions81 model with the ECCw2024 water model, and (b) the Madrid2019 ion model with the TIP4P/2005 water model.

4.3 Calcium

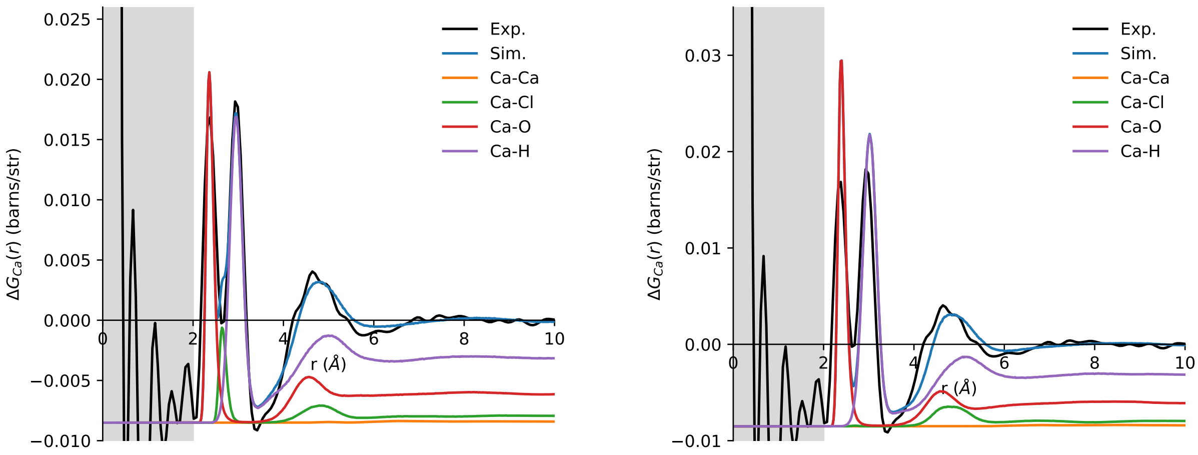

(a) ECCions81-ECCw2024

(b) Madrid2019-TIP4P/2005

Figure S7: First-order difference functions $\Delta G_{Ca}(r)$ and the weighted components for simulations of 4 m $CaCl_2$ solution using (a) the ECCions81 model with the ECCw2024 water model, and (b) the Madrid2019 ion model with the TIP4P/2005 water model.

4.4 Potassium

To further investigate the differences between experimental data and simulations with these ion models in r-space, a parameter set from the optimization process that accurately reproduces the first dual peaks in both position and height was selected as a reference (Figure S8c), with parameters shown in Table S9. This reference model, however, was not chosen as the optimal model by the cost function (Equation 8) due to its poor performance in reproducing the density at 4 m. Specifically, the model predicted a density of 1101.75 kg/m^3, compared to the reference value of 1151.34 kg/m^3 (at 300 K).

$$A(\boldsymbol{\theta}, \boldsymbol{\theta'}) = min\left(1, e^{J(\boldsymbol{\theta'}) - J(\boldsymbol{\theta})}\right) \tag{8}$$

A comparison of K-O and K-Cl radial distribution functions (RDFs) for ECC and Madrid2019 models is shown in Figure S9. Madrid2019 exhibits a higher first peak in both K-O and K-H correlations, closely matching the first minimum and subsequent structure of

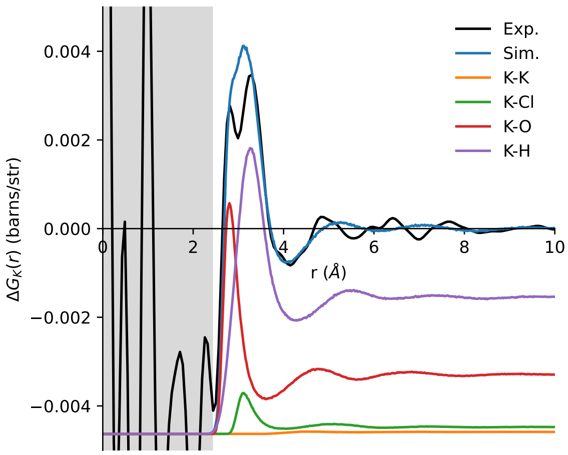

(a) ECCions81-ECCw2024

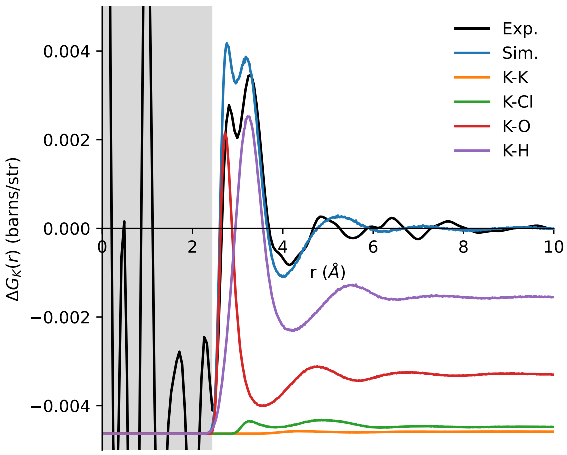

(b) Madrid2019-TIP4P/2005

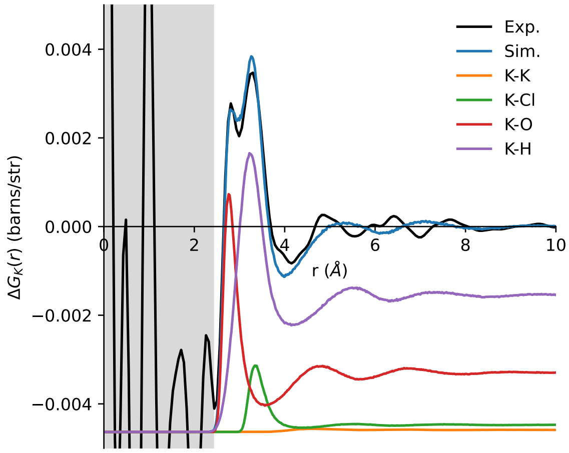

(c) Reference

Figure S8: First-order difference functions, $\Delta G_K(r)$, and their weighted components for simulations of a 6 m LiCl solution using (a) the ECCions81 model with the ECCw2024 water model, (b) the Madrid2019 ion model with the TIP4P/2005 water model, and (c) reference K^+ and Cl^- models with the ECCw2024 water model.

the reference model. On the other hand, ECC produces first peaks in K-O and K-H RDFs similar in height to the reference but with slightly higher first minima and broader peaks due to these elevated minima. For the K-Cl pairing, the reference model shows a strong first peak with a height of 9, while Madrid2019 produces a much smaller first peak (2) and a more pronounced second peak. ECC falls between these extremes, with a moderate first peak height of 6 and a second peak between the reference and Madrid2019. The stronger K-Cl pairing in both the reference model and the ECC model, together with the larger

σ_{KO}, contributes to the lower peaks of correlation between K^+ and water. Additionally, the ECC model shows a shorter K-Cl distance, as evidenced by the earlier onset of the first peak. This shorter K-Cl distance, combined with the broader K-O first peak, causes the dual peaks to merge into a single peak. Overall, in r-space, the ECC model behaves as an intermediate between the reference model (which reproduces the first peak well but fails in density) and Madrid2019 (which struggles to capture the first peak but performs well in density reproduction).

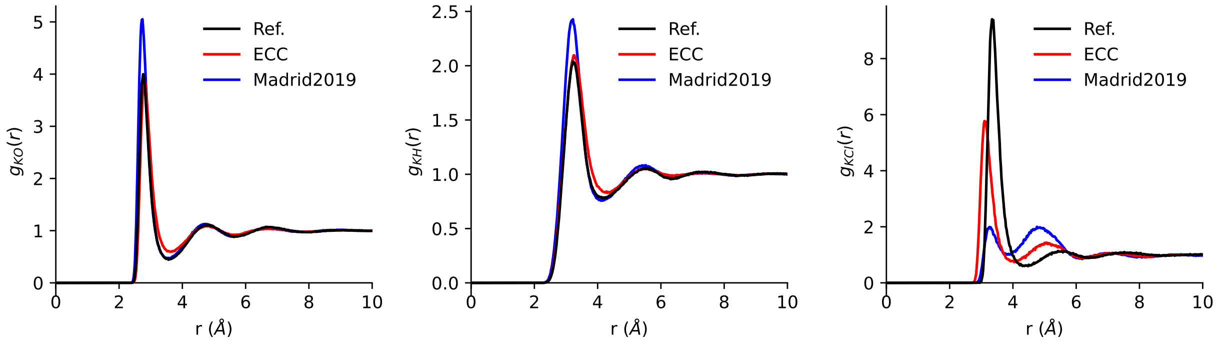

Figure S9: Comparison of radial distribution functions for K-O (left), K-H (middle) and K-Cl (right) in simulations of a 4 m KCl solution. Results are shown for the ECCions81 model with the ECCw2024 water model (red), the Madrid2019 ion model with the TIP4P/2005 water model (blue), and reference models with the ECCw2024 water model (black).

Table S9: Parameters of reference K^+, Cl^- and the small Li^+ models.

**Ion**	σ **(nm)**	ϵ **(kJ/mol)**	**Charges** (e)
Ref KCl			
K^+	0.290037	0.964578	0.86
Cl^-	0.431468	1.650965	-0.86
Cl-O	0.373474	1.120998	
Small Li^+			
Li	0.081675	1.064675	0.81

4.5 Sodium

Figure S10 presents a comparison between simulations and neutron diffraction results for $\Delta G_{KNa}(r)$ and $\Delta Q_{KNa}(r)$. From the comparison in Figures S8 and S11, the main minima

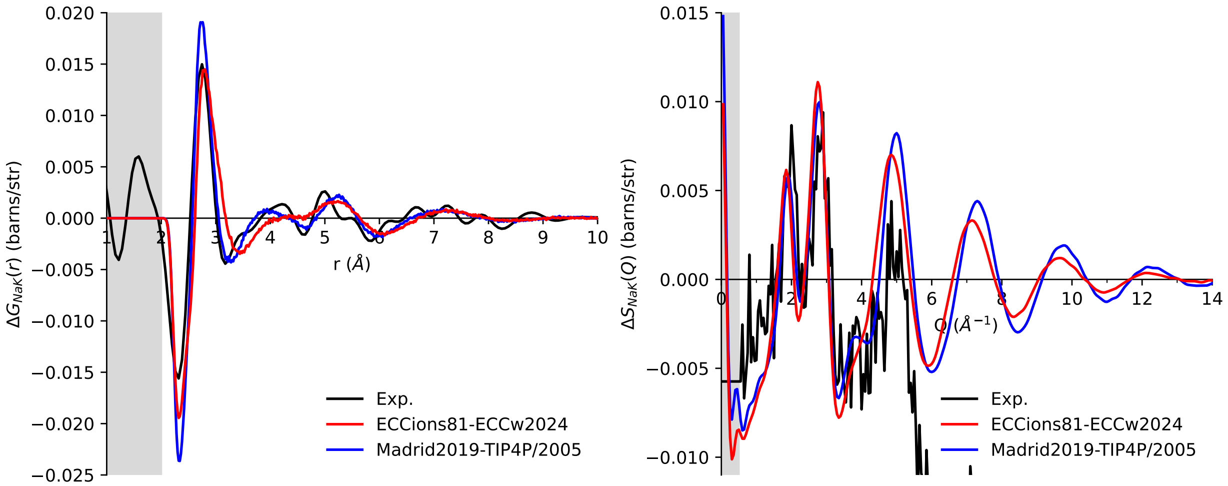

Figure S10: First-order difference functions $\Delta G_{KNa}(r)$ (left) and $\Delta S_{KNa}(Q)$ (right) for simulations of 4 m NaCl and 4 m KCl solutions using the ECCions81 model with the ECCw2024 water model (red), the Madrid2019 ion model with the TIP4P/2005 water model (blue), and experimental data (black).

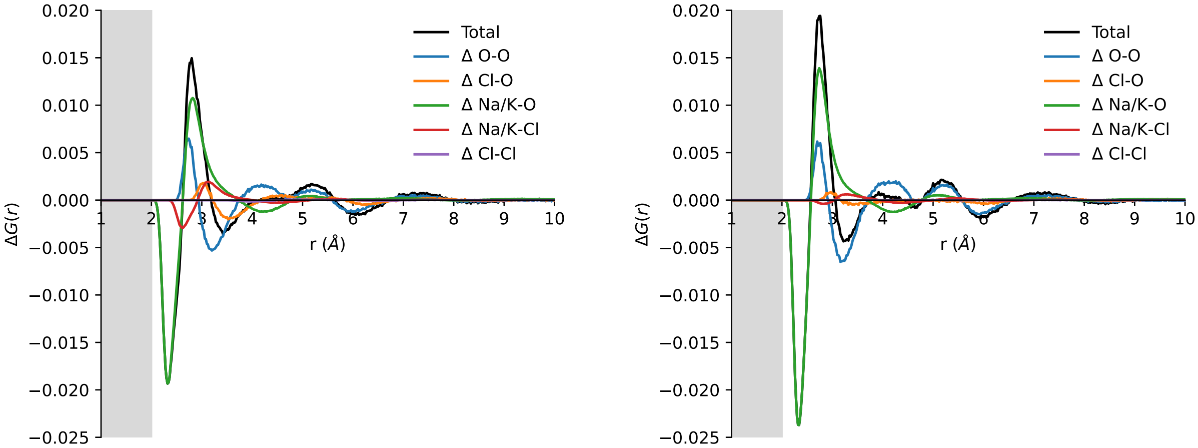

(a) ECCions81-ECCw2024 (b) Madrid2019-TIP4P/2005

Figure S11: First-order difference functions $\Delta G_{KNa}(r)$ and the weighted components for simulations of 4 m KCl and 4 m NaCl solutions using (a) the ECCions81 model with the ECCw2024 water model, and (b) the Madrid2019 ion model with the TIP4P/2005 water model.

in r-space is attributed to the Na-O correlation, while the primary peak is associated with K-O and O-O correlations. ECCions81 predicts a lower amplitude for the Na-O minima compared to Madrid2019, aligning more closely with the experimental data. Additionally, the negative peak at 2.7 Å in the red line of Figure S11a originates from Na-Cl pairing, which

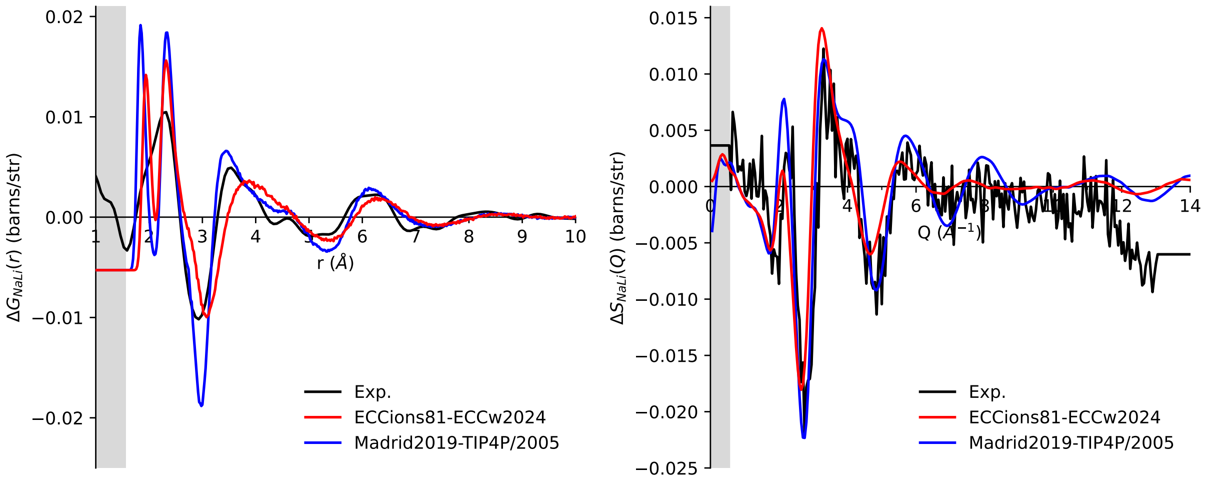

Figure S12: irst-order difference functions $\Delta G_{NaLi}(r)$ (left) and $\Delta S_{NaLi}(Q)$ (right) for simulations of 4 m NaCl and 4 m LiCl solutions using the ECCions81 model with the ECCw2024 water model(red), the Madrid2019 ion model with the TIP4P/2005 water model (blue), a "small" Li^+ model and the ECCions81 Na^+, Cl^- models with the ECCw2024 water model(orange), and experimental data (black).

is absent in Figure S11b. This observation underscores the fact that the ECCions81 model introduces Na-Cl interactions, which are not seen in Madrid2019, resulting in a reduced Na-O valley height. For the combined K-O and O-O peak, ECCions81 predicts a peak height that matches the experimental data but with a broader peak width. This behavior is consistent with the results shown in Figure S9 and aligns with the discussion in the section on Potassium Chloride.

Figure S12 compares the experimental r-space and Q-space signals with those calculated from simulations, alongside the weighted components of specific correlations shown in Figure S13. In r-space, both ECC and Madrid2019 exhibit two distinct peaks between 1.8 and 2.7 Å, whereas the experimental signal shows a single combined peak in this range. Notably, the peak heights predicted by ECC align more closely with the experimental data. By comparing Figure S13 with Figures S3 and S5, it is evident that the first peak originates solely from Li-O correlations, while the second peak is mainly due to Na-O correlations. The relative peak heights compared with the experimental data are consistent with the results observed in $\Delta G_{Li}(r)$ and $\Delta G_{KNa}(r)$. For the main minimum around 3 Å, ECC closely matches

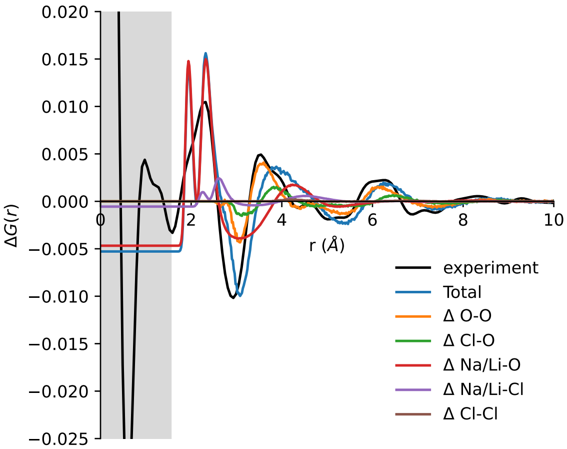

(a) ECCions81-ECCw2024

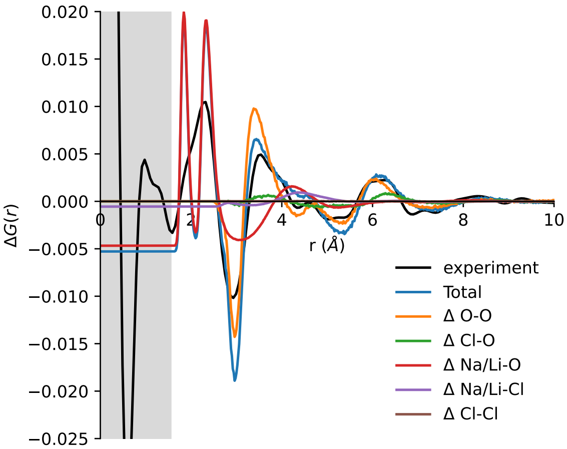

(b) Madrid2019-TIP4P/2005

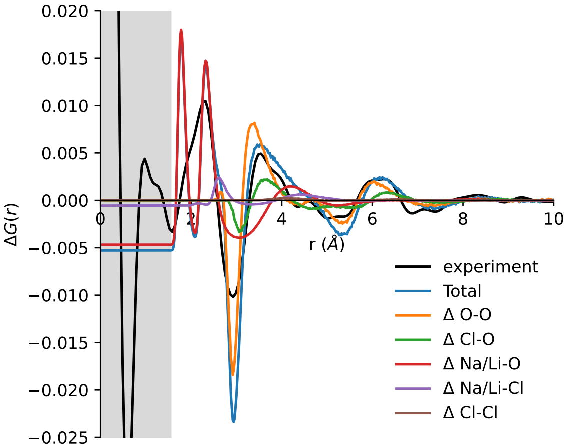

(c) Small Li-ECCw2024

Figure S13: First-order difference functions, $\Delta G_{NaLi}(r)$, and their weighted components for simulations of 4 m LiCl and 4 m NaCl solutions using: (a) the ECCions81 model with the ECCw2024 water model, (b) the Madrid2019 ion model with the TIP4P/2005 water model, and (c) the ECCions81 Na^+, Cl^- ions and a ”small” ECC Li^+ model which has a reduced σ, with the ECCw2024 water model.

the experimental depth, though it is shifted by approximately 0.2 Å. On the other hand, Madrid2019 captures the position more accurately but overestimates the depth, showing twice the amplitude of the experimental signal. Beyond 3 Å, ECC exhibits a significant phase shift compared to the experimental data, while Madrid2019 provides a better fit in this region. In Q-space, ECC demonstrates comparable agreement with experimental values below 4 $Å^{-1}$. However, above 4 $Å^{-1}$, a noticeable phase shift and deviations in peak heights

are observed, whereas Madrid2019 maintains better agreement with the experimental data in the whole range.

To identify the source of deviations in the ECC model results, we introduced a modified ECC Li^+ ion with a smaller σ (Table S9) and simulated a 4 m LiCl solution using this modified ion alongside the ECC Cl^-. The resulting signal, subtracted from the ECC NaCl signal, is shown as the yellow line in Figure S12. This modified Li^+ model produced results similar to Madrid2019 in both r-space and Q-space, with slightly larger amplitudes for peaks and minima in both spaces. These findings indicate that the deviations originate from the Li^+ model rather than the Na^+ or Cl^- models, confirming that the ECC Na^+ model is compatible with Madrid2019 in reproducing neutron diffraction data. Additionally, this comparison helps clarify the effects arising from differences in Li^+ models. By analyzing the components in Figures S22a and S13c, we determined that the primary differences stem from O-O correlations in the 2.8–6 Å range. While the ECC Li^+ ion performs well in reproducing NDIS results, which focus on Li-water, and Li-Cl correlations with O-O contributions canceled out during isotope substitution, it falls short in accurately capturing its impact on O-O correlations.

4.6 Magnesium

Concerning the $MgCl_2$ solution, it is important to note that the water exchange rate in the first hydration shell of Mg^{2+} is very low—on the order of milliseconds for a single exchange event. Given the limited simulation timescales used during the optimization, it is clear that the sampling is insufficient to fully capture such slow exchange dynamics. Since all simulations, including those using the Madrid2019 parameters, began from the same initial structure but ultimately stabilized in different configurations, we assume that the system relaxes into a locally stable structure within the sampled timescale. However, this outcome should be interpreted with caution due to potential undersampling effects.

To further support the reliability of our findings, we conducted three independent 10 ns

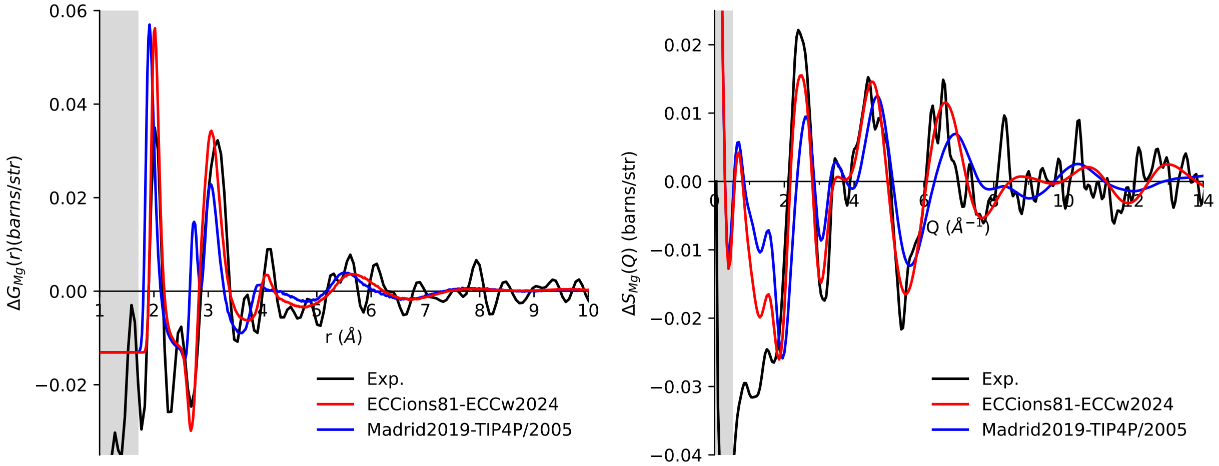

Figure S14: First-order difference functions $\Delta G_{MgW}(r)$ (left) and $\Delta S_{MgW}(Q)$ (right) for simulations of a 3 m $MgCl_2$ solution using the ECCions81 model with the ECCw2024 water model (red), the Madrid2019 ion model with the TIP4P/2005 water model (blue), and experimental data (black).

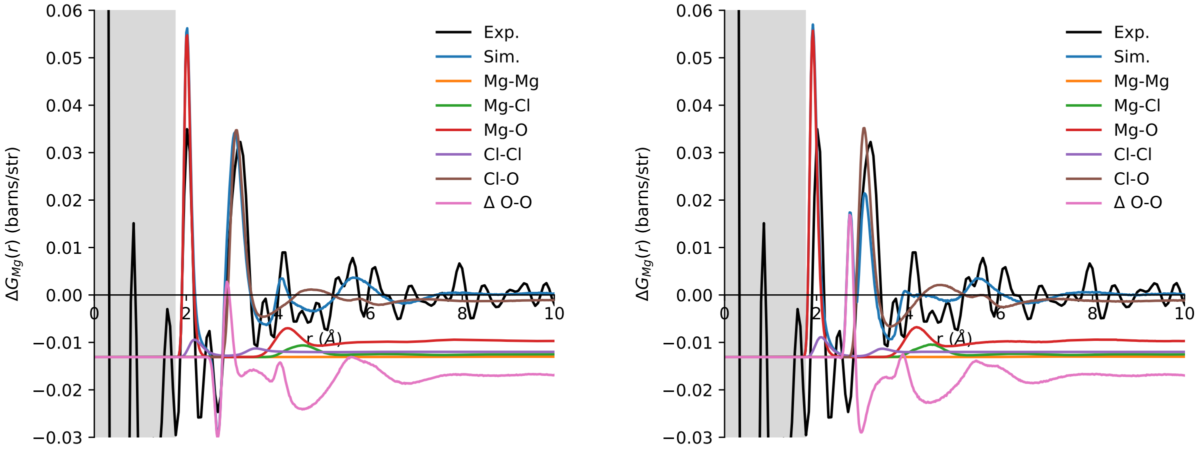

(a) ECCions81-ECCw2024

(b) Madrid2019-TIP4P/2005

Figure S15: First-order difference functions $\Delta G_{MgW}(r)$ and the weighted components for simulations of a 3 m $MgCl_2$ solution using (a) the ECCions81 model with the ECCw2024 water model, and (b) the Madrid2019 ion model with the TIP4P/2005 water model.

simulations of a 3 m $MgCl_2$ solution starting from two distinct initial configurations: one with 4 contact Mg–Cl ion pairs (init-0) and another with 77 pairs (init-1), each system containing 150 Mg^{2+} and 300 Cl^- ions (Figure S16). As shown in Figure S17, the Mg–O distributions were similar across both configurations, while the Mg–Cl RDFs exhibited marked differences—init-1 displayed a significantly higher first peak, reflecting the higher number of

contact pairs in the initial structure. To assess equilibration, one replica from each configuration was extended to 500 ns, and the potential energy profiles confirmed that both systems reached equilibrium after ∼200 ns (Figure S18). Eventually, both simulations converged to nearly identical Mg–O and Mg–Cl RDFs: the Mg–Cl RDFs showed a dramatic reduction in the first peak, while the Mg–O RDFs exhibited a slight increase(Figure S17). This indicates that, over long timescales, the RDFs become independent of the initial ion-pairing configuration and represent an equilibrated state, which we used for the results reported in the main text. For reproducibility, three independent 500 ns NPT simulations were performed for 4 m $MgCl_2$. As in the 3 m $MgCl_2$ case, the potential energy profiles indicate that the system requires at least 150 ns to reach equilibration (Figure S19). RDFs calculated after discarding the first 150 ns (Figure S20) show similar behavior to those obtained for the 3 m $MgCl_2$ system. CIP)value for the 4 m $MgCl_2$ solution was then calculated as the average of the three replicas.

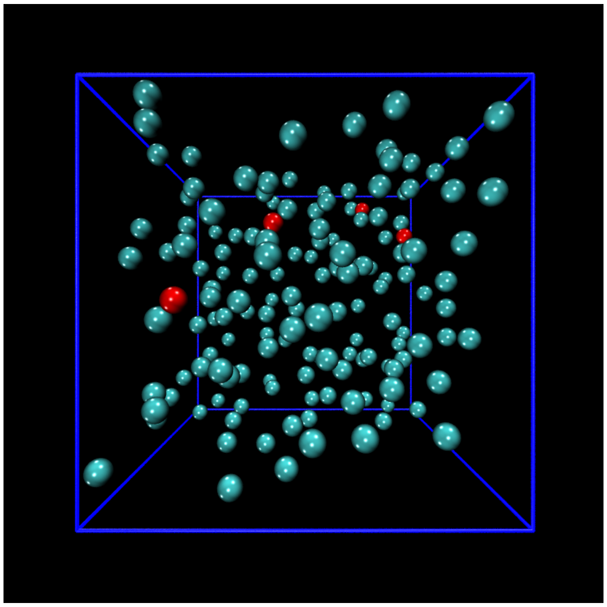

(a) initial-0: 4 contact pairs

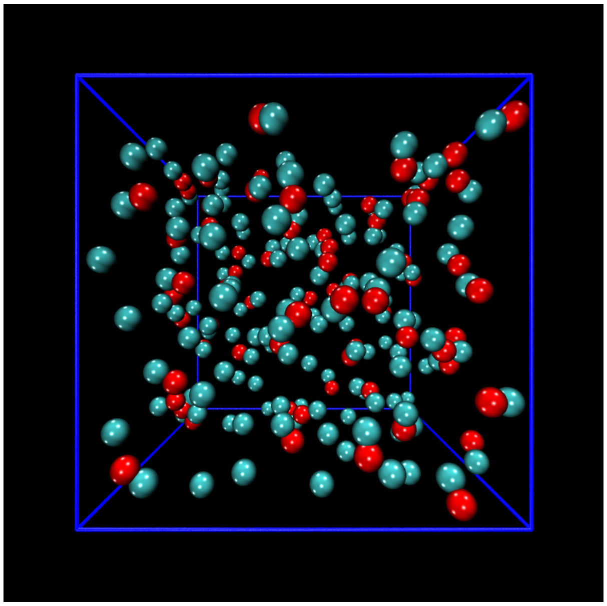

(b) initial-1: 77 contact pairs

Figure S16: Two initial configurations of a 3 m $MgCl_2$ solution. Cyan spheres represent Mg^{2+} ions and red spheres represent Cl^- ions; only contact-paired Cl^- ions are shown, with water molecules omitted for clarity. (a) Init-0: 4 Mg^{2+}–Cl^- contact pairs; (b) init-1: 77 Mg^{2+}–Cl^- contact pairs.

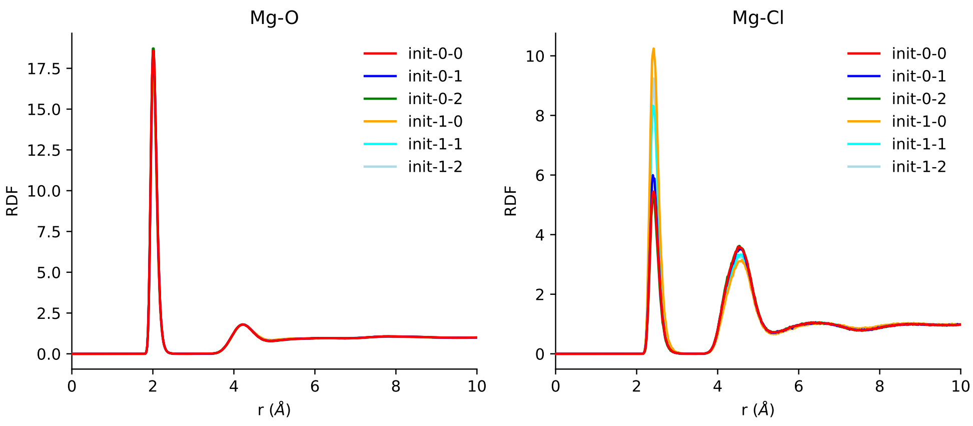

(a) Short(10 ns) simulations.

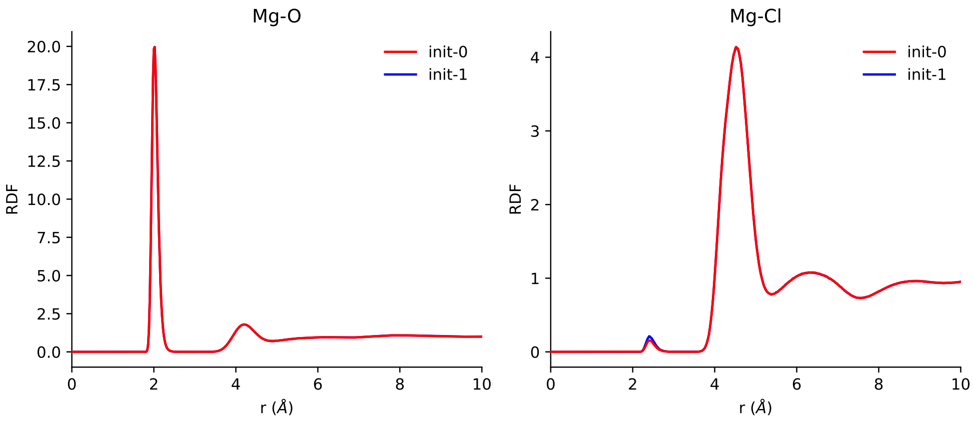

(b) Long(500 ns) simulations.

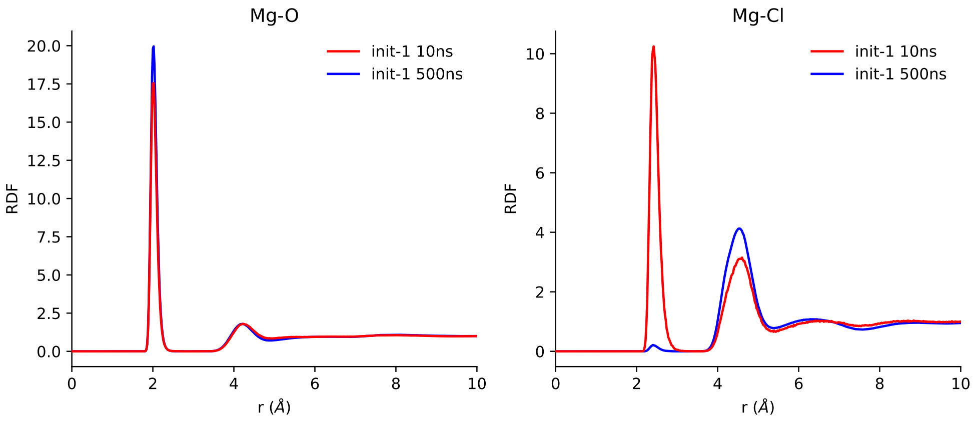

(c) Short/Long

Figure S17: Comparison of Mg–O(left) and Mg–Cl(right) RDFs from simulations starting with different initial configurations of a 3 m $MgCl_2$ solution. (a) 10 ns simulations from init-0 and init-1 show distinct RDFs; After 500 ns, the RDFs converge; (c) Comparison between 10 ns and 500 ns results.

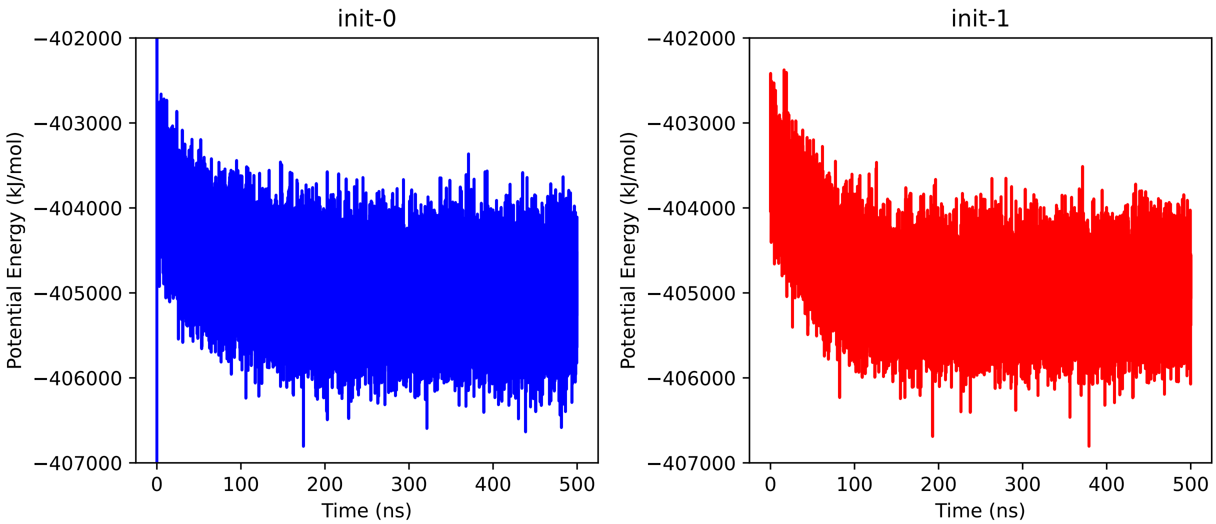

Figure S18: Time series of the potential energy for 3 m $MgCl_2$ systems initialized from init-0(left) and init-1(right) configurations.

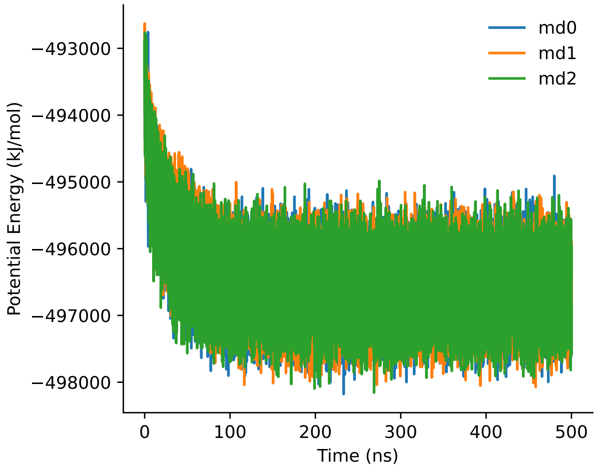

Figure S19: Time series of the potential energy for three independent simulations of 4 m $MgCl_2$ systems, each initialized from the same configuration.

The long equilibration times are attributed to the slow exchange rates of water and Cl^- in the first hydration shell of Mg^{2+}. This slow relaxation was not observed in simulations with the Madrid2019 parameters, which were designed with strong Mg–Cl repulsion to prevent ion pairing. As a result, even simulations starting from init-1 rapidly lost contact pairs during energy minimization and did not undergo prolonged Cl^-/water exchange processes. Notably, the equilibration primarily involved Mg–Cl reorganization relative to Mg–water coordination,

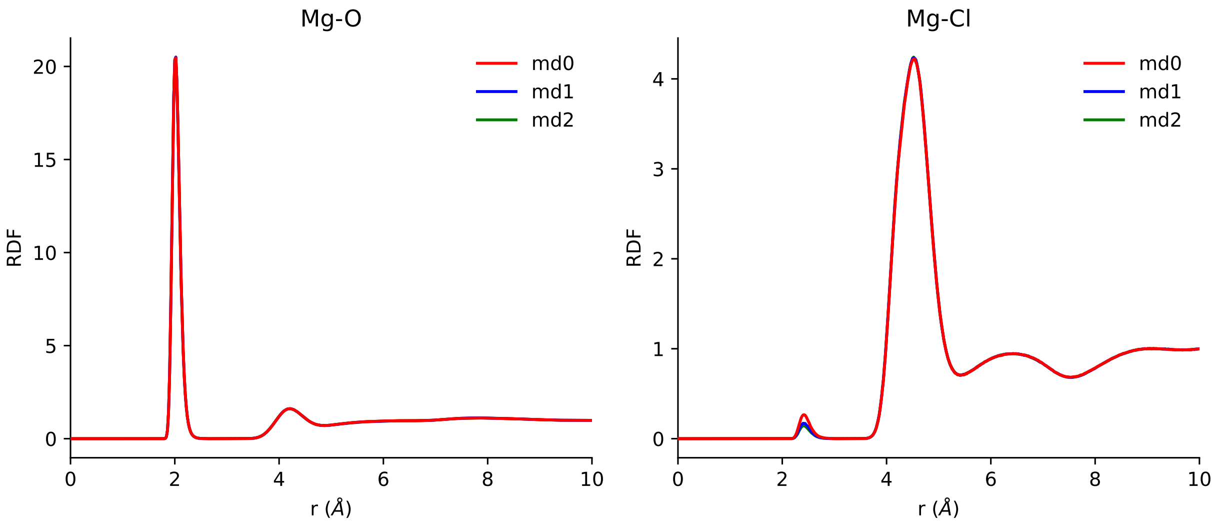

Figure S20: Comparison of Mg–O(left) and Mg–Cl(midd) RDFs from three independent simulations initialized from the same initial configurations of a 4 m $MgCl_2$ solution.

with no water exchange events observed within the Mg^{2+} first hydration shell—highlighting a potential undersampling of Mg–Cl and Mg–water partitioning. Future work will address this limitation through enhanced sampling techniques such as replica exchange molecular dynamics.

4.7 Bromide

For KBr, in Q-space, ECCions81 and Madrid2019 exhibit similar phase reproduction, both showing a broader first peak compared to the experiment. ECCions81 predicts a higher peak at 2.3 $Å^{-1}$, closer to the experimental data, though both models still underestimate the amplitudes of peaks and valleys beyond 2 $Å^{-1}$. Below 1.5 $Å^{-1}$, both models show lower intensities than the experimental signal.

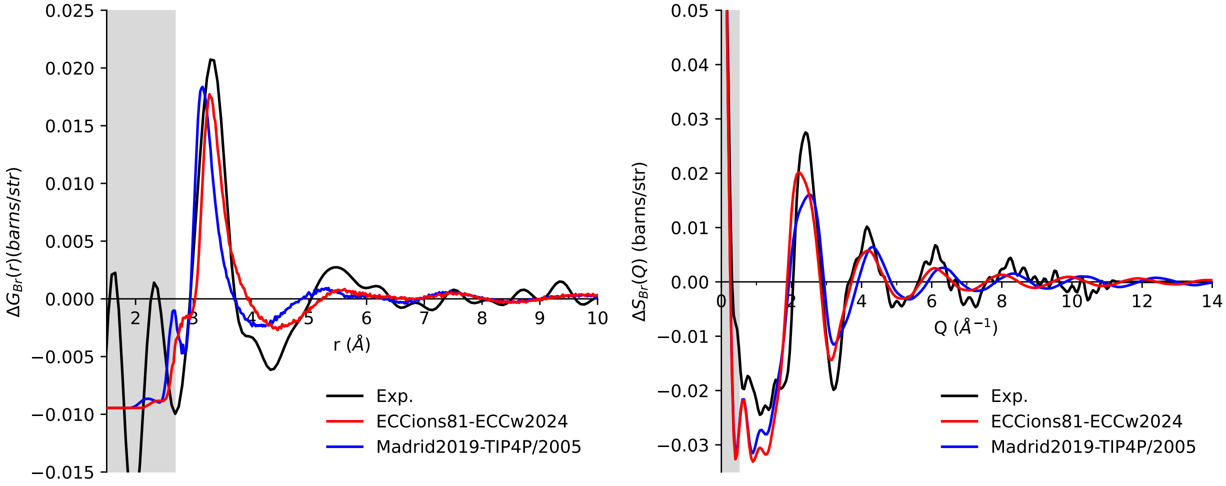

Figure S21: First-order difference functions $\Delta G_{BrW}(r)$ (left) and $\Delta S_{BrW}(Q)$ (right) for simulations of a 4 m KBr solution using the ECCions81 model with the ECCw2024 water model(red), the Madrid2019 ion model with the TIP4P/2005 water model (blue), and experimental data (black).

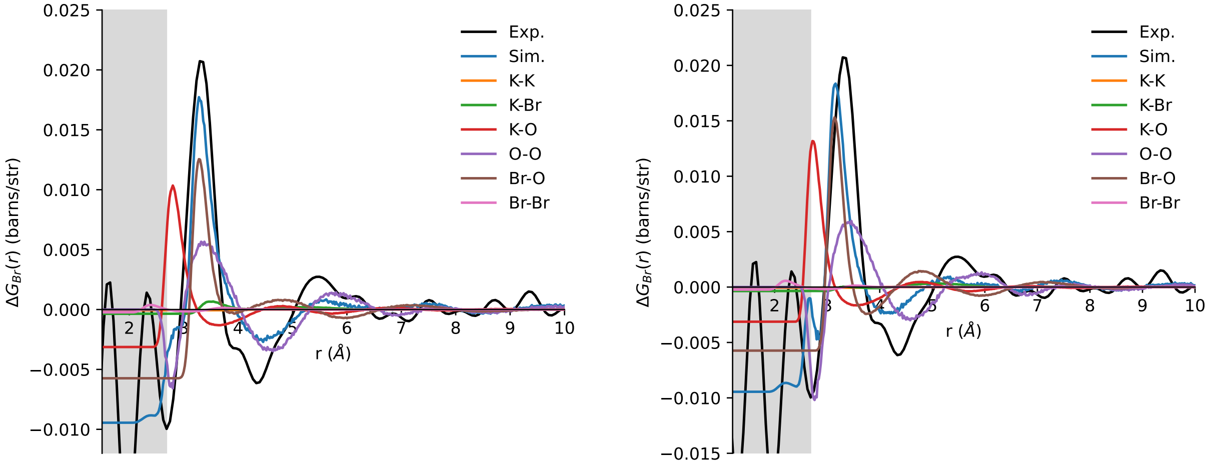

(a) ECCions81-ECCw2024 (b) Madrid2019-TIP4P/2005

Figure S22: First-order difference functions $\Delta G_{BrW}(r)$ and the weighted components for simulations of a 4 m KBr_2 solution using (a) the ECCions81 model with the ECCw2024 water model, and (b) the Madrid2019 ion model with the TIP4P/2005 water model.

4.8 Iodide

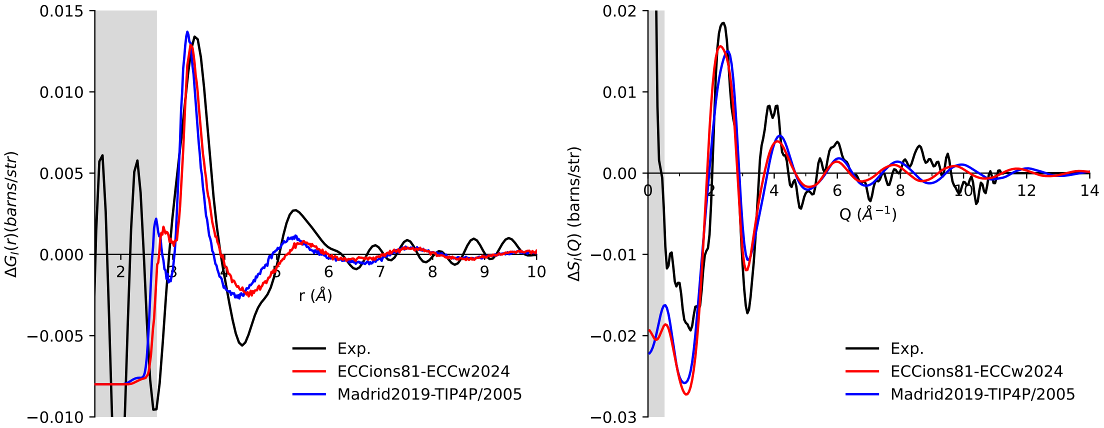

Figure S23: First-order difference functions $\Delta G_{IW}(r)$ (left) and $\Delta S_{IW}(Q)$ (right) for simulations of a 4 m KI solution using ECCions81 models with the ECCw2024 water model (red), Madrid2019 ion models with the TIP4P/2005 water model (blue), and experimental data (black).

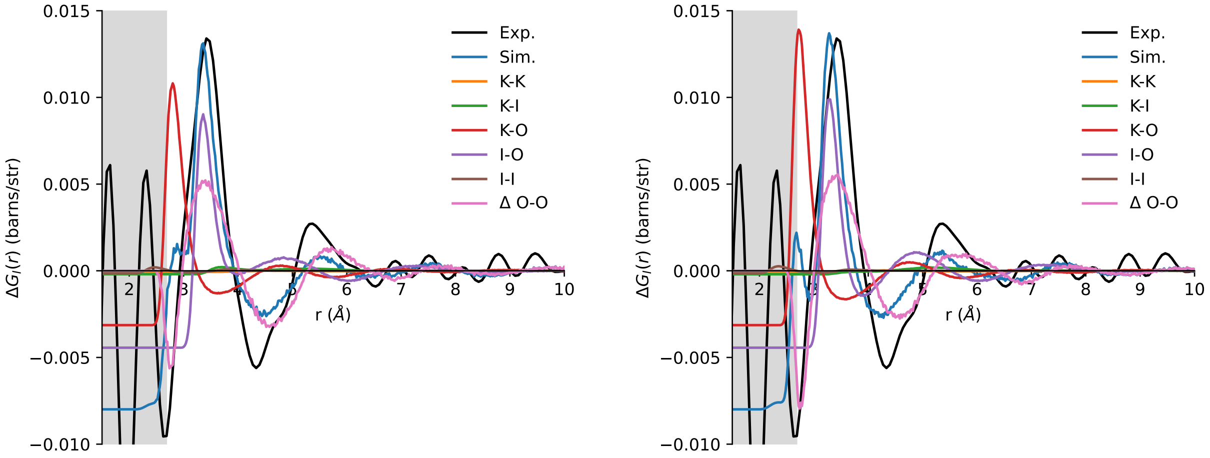

(a) ECCions81-ECCw2024

(b) Madrid2019-TIP4P/2005

Figure S24: First-order difference functions $\Delta G_{IW}(r)$ and the weighted components for simulations of a 4 m KI_2 solution using (a) the ECCions81 model with the ECCw2024 water model, and (b) the Madrid2019 ion model with the TIP4P/2005 water model.

For KI, in Q-space ECCions81 shows better agreement with experiments than Madrid2019 in the amplitude of the first peak at 2.3 $Å^{-1}$. and the first valley at 3.1 $Å^{-1}$. Both models capture the experimental phases well between 2 and 7 $Å^{-1}$, though they continue to underestimate the amplitudes of peaks and valleys beyond 2 $Å^{-1}$.

5 Water effect in physical and structural properties for ECCions81

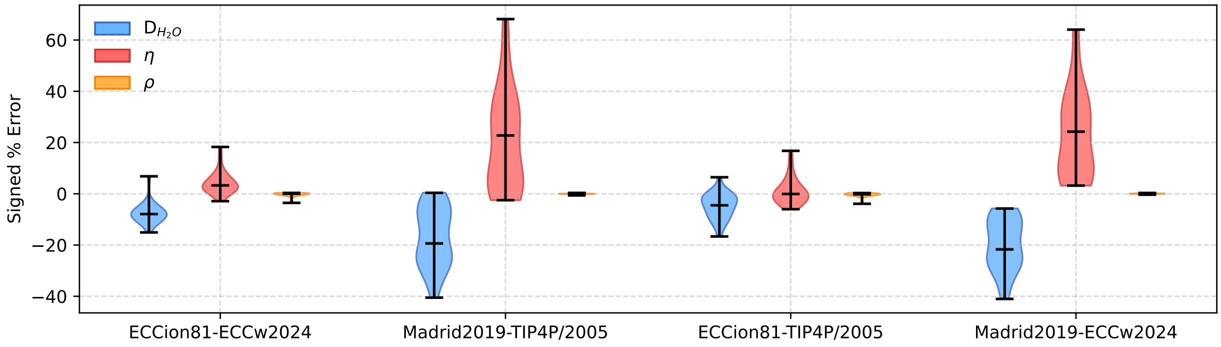

Figure S25: Signed percentage errors in the self-diffusion coefficient of water ($D_{\mathrm{H_2O}}$), viscosity (η), and density (ρ) predicted from simulations using four water-ion models: ECCions81-ECCw2024, Madrid2019-TIP4P/2005, ECCions81-TIP4P/2005, and Madrid2019-ECCw2024, with experimental data as the reference. Violin plots represent the distribution of errors across all tested electrolyte solutions at various concentrations. Horizontal bars indicate the medians, and the full range of data is shown to highlight variability and systematic deviations.

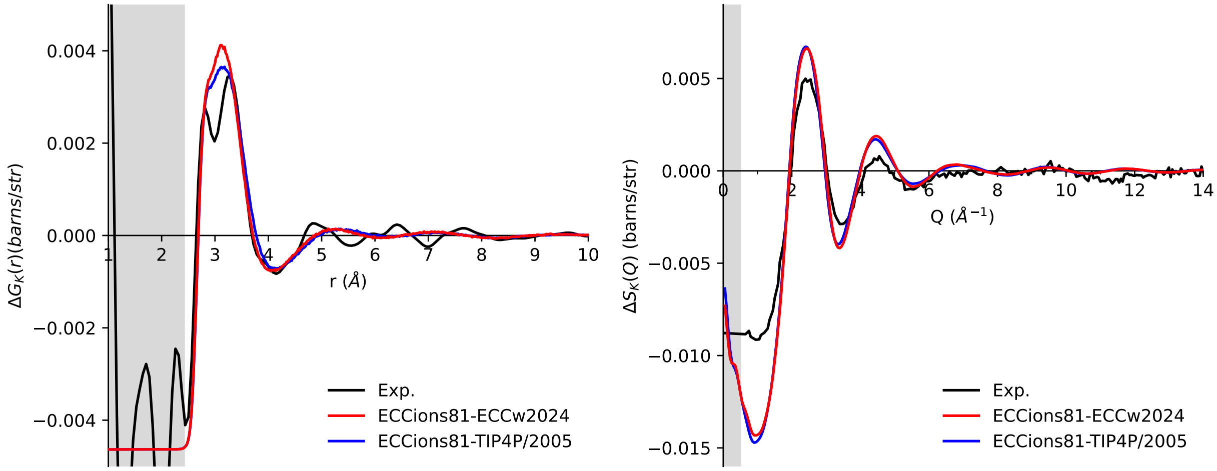

Figure S26: First-order difference functions $\Delta G_K(r)$ (left) and $\Delta S_K(Q)$ (right) for simulations of a 4 m KCl solution using the ECCions81 model combined with either the ECCw2024 water model (red) or the TIP4P/2005 water model (blue), compared to experimental data (black).

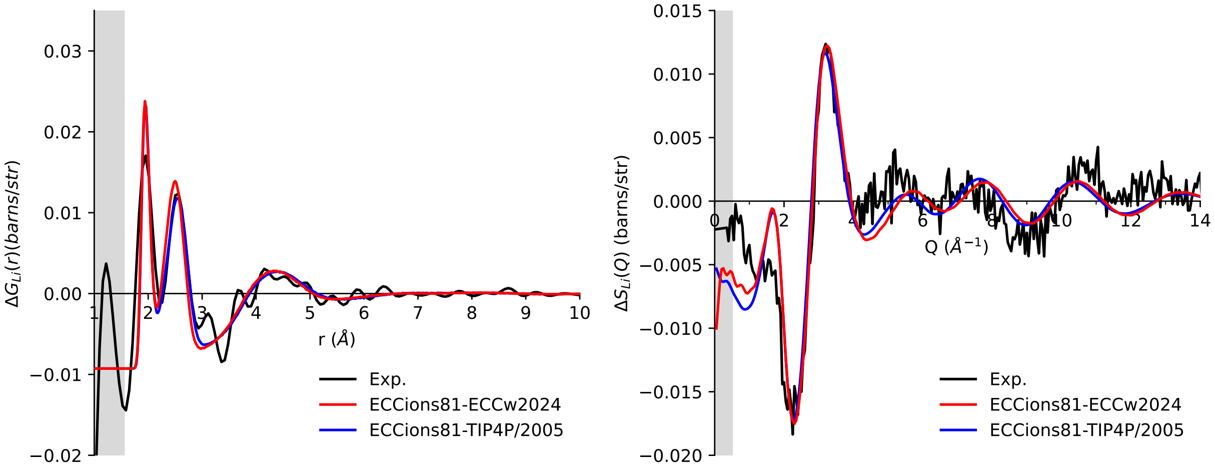

Figure S27: First-order difference functions $\Delta G_{Li}(r)$ (left) and $\Delta S_{Li}(Q)$ (right) for simulations of a 3 m LiCl solution using the ECCions81 model combined with either the ECCw2024 water model (red) or the TIP4P/2005 water model (blue), compared to experimental data (black).

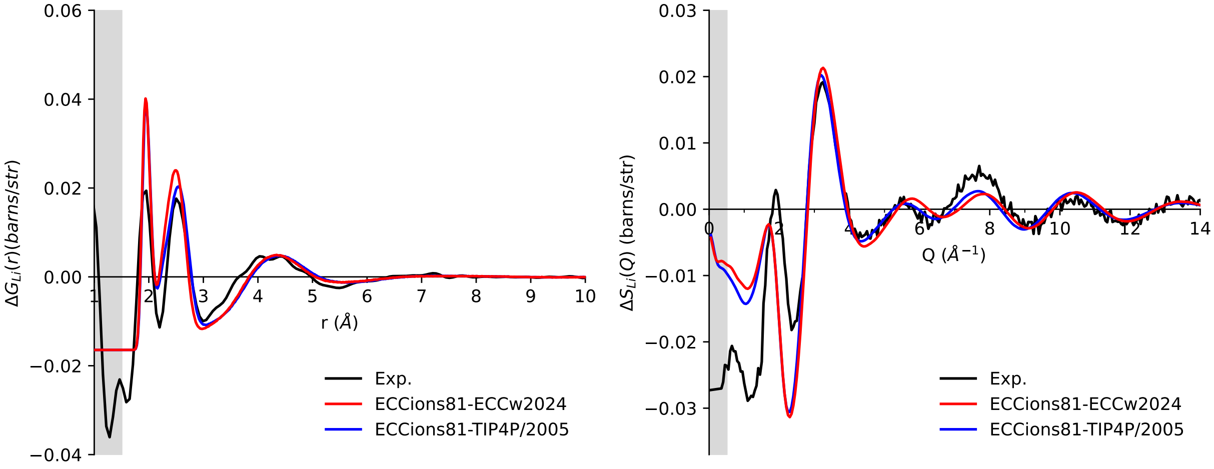

Figure S28: First-order difference functions $\Delta G_{Li}(r)$ (left) and $\Delta S_{Li}(Q)$ (right) for simulations of a 6 m LiCl solution using the ECCions81 model combined with either the ECCw2024 water model (red) or the TIP4P/2005 water model (blue), compared to experimental data (black).

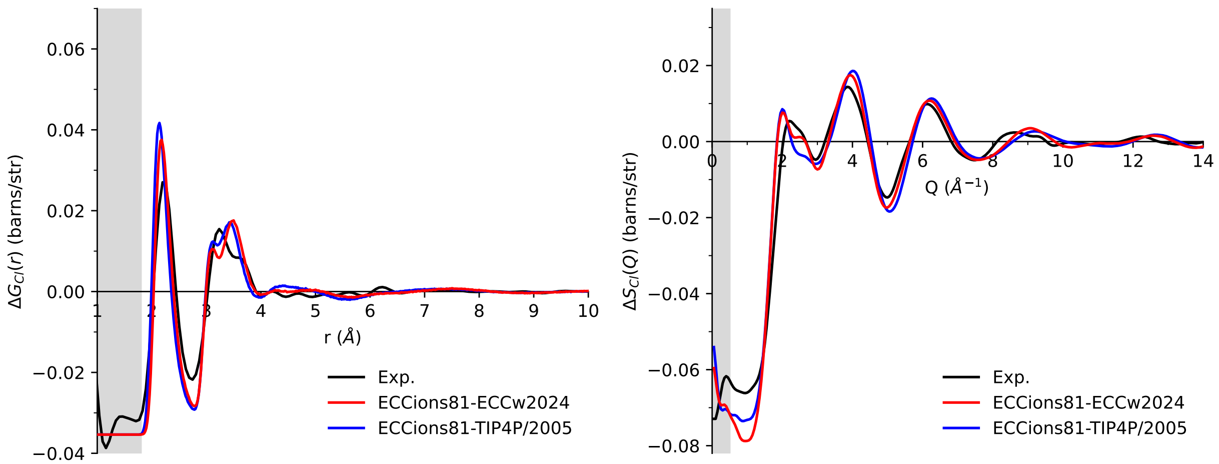

Figure S29: First-order difference functions $\Delta G_{Cl}(r)$ (left) and $\Delta S_{Cl}(Q)$ (right) for simulations of a 6 m LiCl solution using the ECCions81 model combined with either the ECCw2024 water model (red) or the TIP4P/2005 water model (blue), compared to experimental data (black).

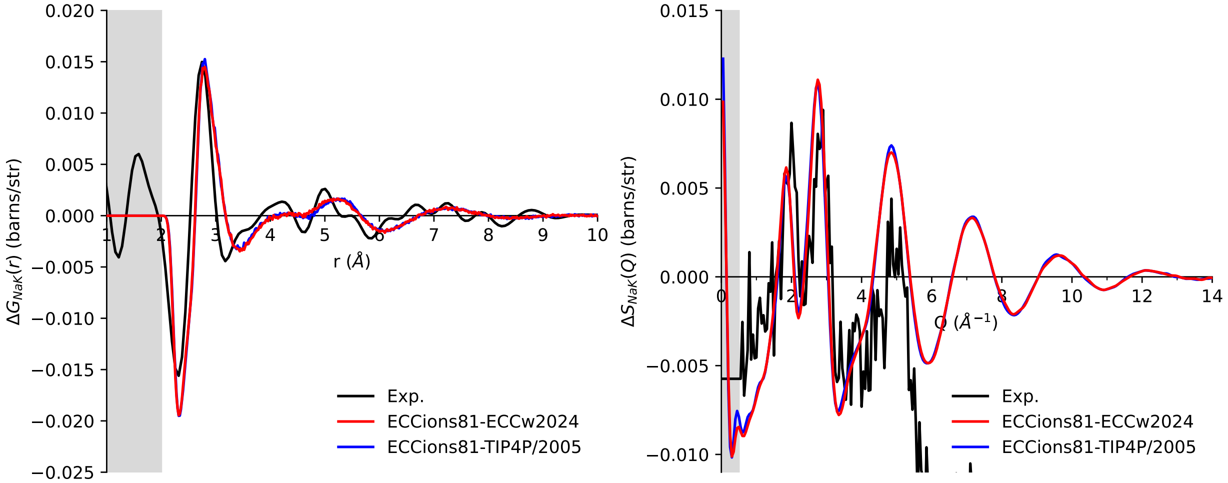

Figure S30: First-order difference functions $\Delta G_{KNa}(r)$ (left) and $\Delta S_{KNa}(Q)$ (right) for simulations of 4 m NaCl and 4 m KCl solutions using the ECCions81 model combined with either the ECCw2024 water model (red) or the TIP4P/2005 water model (blue), compared to experimental data (black).

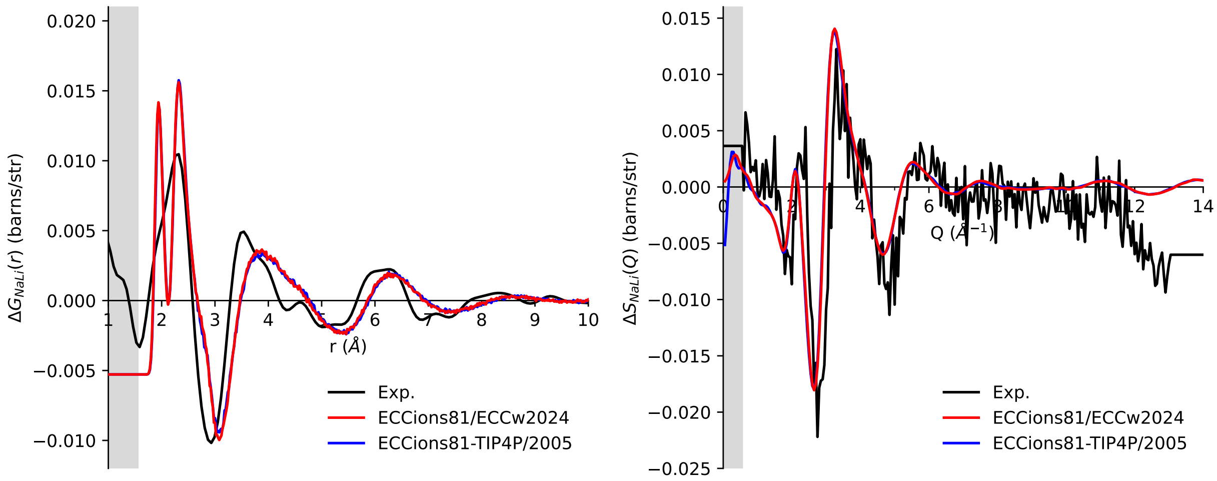

Figure S31: First-order difference functions $\Delta G_{NaLi}(r)$ (left) and $\Delta S_{NaLi}(Q)$ (right) for simulations of 4 m NaCl and 4 m LiCl solutions using the ECCions81 model combined with either the ECCw2024 water model (red) or the TIP4P/2005 water model (blue), compared to experimental data (black).

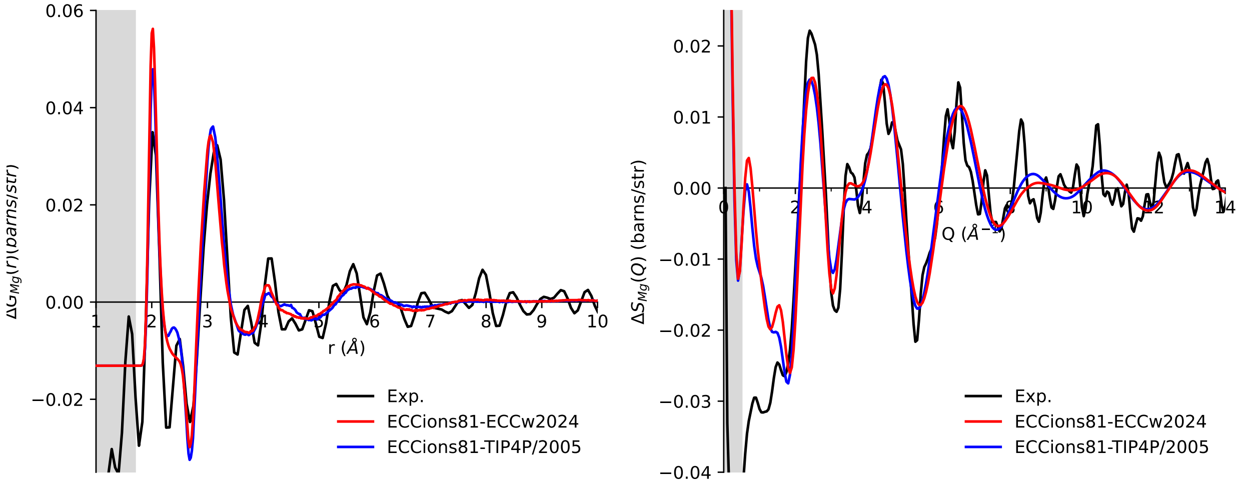

Figure S32: First-order difference functions $\Delta G_{MgW}(r)$ (left) and $\Delta S_{MgW}(Q)$ (right) for simulations of a 3 m $MgCl_2$ solution using the ECCions81 model combined with either the ECCw2024 water model (red) or the TIP4P/2005 water model (blue), compared to experimental data (black).

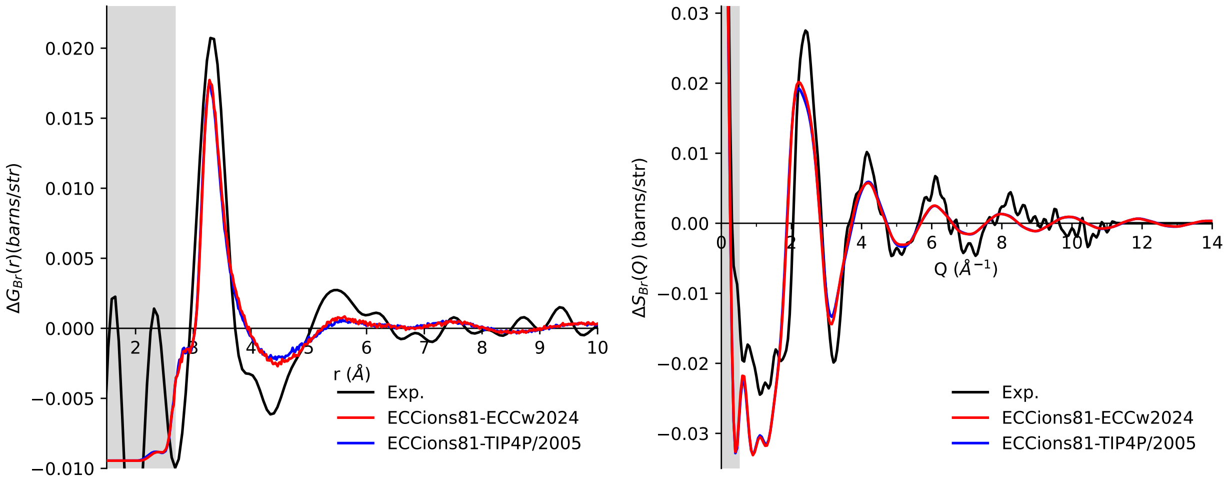

Figure S33: First-order difference functions $\Delta G_{BrW}(r)$ (left) and $\Delta S_{BrW}(Q)$ (right) for simulations of a 4 m KBr solution using the ECCions81 model combined with either the ECCw2024 water model (red) or the TIP4P/2005 water model (blue), compared to experimental data (black).

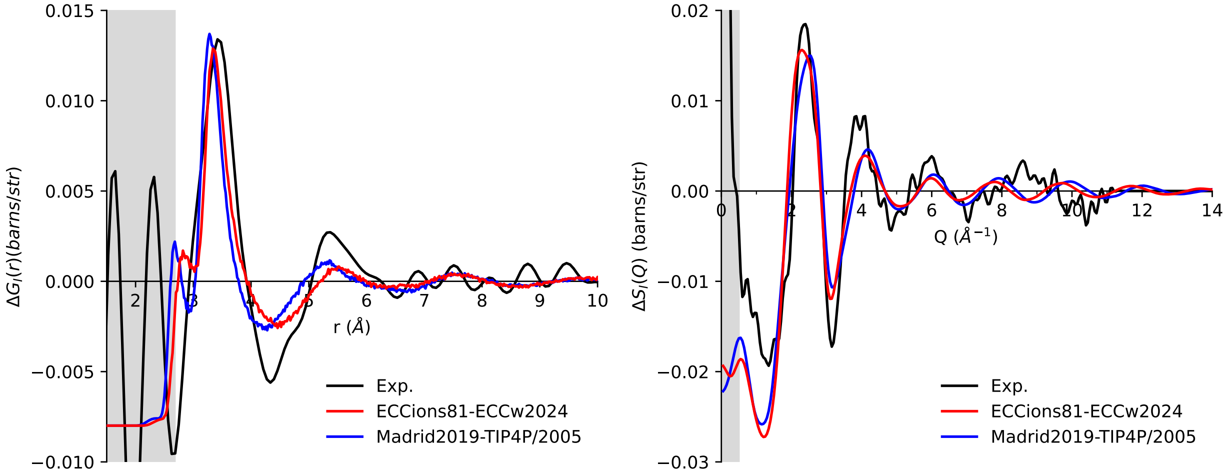

Figure S34: First-order difference functions $\Delta G_{IW}(r)$ (left) and $\Delta S_{IW}(Q)$ (right) for simulations of the a KI solution using the ECCions81 model combined with either the ECCw2024 water model (red) or the TIP4P/2005 water model (blue), compared to experimental data (black).

References

(S1) Blazquez, S.; Conde, M. M.; Abascal, J. L. F.; Vega, C. The Madrid-2019 force field for electrolytes in water using TIP4P/2005 and scaled charges: Extension to the ions F-, Br-, I-, Rb+, and Cs. *156*, 044505.

(S2) Dočkal, J.; Mimrová, P.; Lísal, M.; Moučka, F. Structure of aqueous alkali metal halide electrolyte solutions from molecular simulations of phase-transferable polarizable models. *394*, 123797.

(S3) Ikeda, T.; Boero, M.; Terakura, K. Hydration of alkali ions from first principles molecular dynamics revisited. *126*, 034501.

(S4) Hofer, T. S. Solvation Structure and Ion–Solvent Hydrogen Bonding of Hydrated Fluoride, Chloride and Bromide—A Comparative QM/MM MD Simulation Study. *2*, 445–464, Number: 4 Publisher: Multidisciplinary Digital Publishing Institute.

(S5) Tongraar, A.; Hannongbua, S.; Rode, B. M. QM/MM MD Simulations of Iodide Ion (I-) in Aqueous Solution: A Delicate Balance between Ion-Water and Water-Water H-Bond Interactions. *114*, 4334–4339, Publisher: American Chemical Society.

(S6) Azam, S. S.; Hofer, T. S.; Randolf, B. R.; Rode, B. M. Hydration of Sodium(I) and Potassium(I) Revisited: A Comparative QM/MM and QMCF MD Simulation Study of Weakly Hydrated Ions. *113*, 1827–1834, Publisher: American Chemical Society.

(S7) Guàrdia, E.; Skarmoutsos, I.; Masia, M. On Ion and Molecular Polarization of Halides in Water. *5*, 1449–1453, Publisher: American Chemical Society.

(S8) Marcus, Y. Effect of Ions on the Structure of Water: Structure Making and Breaking. *109*, 1346–1370, Publisher: American Chemical Society.

(S9) Ohtaki, H.; Radnai, T. Structure and dynamics of hydrated ions. *93*, 1157–1204, Publisher: American Chemical Society.

(S10) Ramos, S.; Barnes, A. C.; Neilson, G. W.; Capitan, M. J. Anomalous X-ray diffraction studies of hydration effects in concentrated aqueous electrolyte solutions. *258*, 171–180.

(S11) Fulton, J. L.; Pfund, D. M.; Wallen, S. L.; Newville, M.; Stern, E. A.; Ma, Y. Rubidium ion hydration in ambient and supercritical water. *105*, 2161–2166.

(S12) Sedano, L. F.; Blazquez, S.; Noya, E. G.; Vega, C.; Troncoso, J. Maximum in density of electrolyte solutions: Learning about ion–water interactions and testing the Madrid-2019 force field. *156*, 154502.

(S13) Gámez, F.; Sedano, L. F.; Blazquez, S.; Troncoso, J.; Vega, C. Building a Hofmeister-like series for the maximum in density temperature of aqueous electrolyte solutions. *377*, 121433.

(S14) Laliberté, M.; Cooper, W. E. Model for Calculating the Density of Aqueous Electrolyte Solutions. *49*, 1141–1151, Publisher: American Chemical Society.
